# Hypoxia-inducible factor-1 alpha as a therapeutic target for primary effusion lymphoma

**DOI:** 10.1371/journal.ppat.1006628

**Published:** 2017-09-18

**Authors:** Prabha Shrestha, David A. Davis, Ravindra P. Veeranna, Robert F. Carey, Coralie Viollet, Robert Yarchoan

**Affiliations:** 1 HIV and AIDS Malignancy Branch, Center for Cancer Research, National Cancer Institute, Bethesda, Maryland, United States of America; 2 The Wellcome Trust Centre for Human Genetics, University of Oxford, Oxford, United Kingdom; University of Pennsylvania Medical School, UNITED STATES

## Abstract

Primary effusion lymphoma (PEL) is an aggressive B-cell lymphoma with poor prognosis caused by Kaposi’s sarcoma-associated herpesvirus (KSHV). Previous studies have revealed that HIF-1α, which mediates much of the cellular response to hypoxia, plays an important role in life cycle of KSHV. KSHV infection promotes HIF-1α activity, and several KSHV genes are in turn activated by HIF-1α. In this study, we investigated the effects of knocking down HIF-1α in PELs. We observed that HIF-1α knockdown in each of two PEL lines leads to a reduction in both aerobic and anaerobic glycolysis as well as lipid biogenesis, indicating that HIF-1α is necessary for maintaining a metabolic state optimal for growth of PEL. We also found that HIF-1α suppression leads to a substantial reduction in activation of lytic KSHV genes, not only in hypoxia but also in normoxia. Moreover, HIF-1α knockdown led to a decrease in the expression of various KSHV latent genes, including LANA, vCyclin, kaposin, and miRNAs, under both normoxic and hypoxic conditions. These observations provide evidence that HIF-1α plays an important role in PEL even in normoxia. Consistent with these findings, we observed a significant inhibition of growth of PEL in normoxia upon HIF-1α suppression achieved by either HIF-1α knockdown or treatment with PX-478, a small molecule inhibitor of HIF-1α. These results offer further evidence that HIF-1α plays a critical role in the pathogenesis of PEL, and that inhibition of HIF-1α can be a potential therapeutic strategy in this disease.

## Author summary

Kaposi’s sarcoma-associated herpesvirus (KSHV) is an oncogenic herpesvirus that causes several malignancies including primary effusion lymphoma (PEL). PEL is an aggressive B-cell lymphoma that usually develops in a hypoxic environment. There is no standard treatment for PEL and it carries a poor prognosis. Previous studies have revealed that certain KSHV-encoded genes are activated by hypoxia-inducible factor 1 (HIF-1), an intracellular factor that mediates much of the cellular response to hypoxia. KSHV in turn can upregulate HIF-1, suggesting HIF-1 might play a substantial role in PEL oncogenesis. Here, we report for the first time the effects of suppressing HIF-1α, an oxygen-sensitive subunit of HIF-1, in PEL tumor cells. We demonstrate that suppressing HIF-1α can dramatically affect the oncogenic metabolic signature of PELs, replication of KSHV, expression of KSHV-encoded oncogenes, and the growth of PEL cells. Findings presented here not only provide new insights into the role of HIF-1α in KSHV-induced tumors but also provide a rationale for using anti-HIF-1α agents as a therapeutic strategy for PEL and potentially other KSHV-associated malignancies.

## Introduction

Primary effusion lymphoma (PEL) is an aggressive B-cell non-Hodgkin’s lymphoma caused by an oncogenic human herpesvirus, Kaposi’s sarcoma-associated herpesvirus (KSHV), also called human herpesvirus-8 (HHV-8). KSHV is also the causative agent of Kaposi sarcoma (KS), a form of multicentric Castleman’s disease (MCD), and KSHV inflammatory cytokine syndrome (KICS) [1–4]. PEL arises almost exclusively in patients with a compromised immune system, particularly those with AIDS. Tumor cells in most cases are also co-infected with Epstein-Barr virus (EBV). There is no standard treatment for PEL. Patients are generally administered combination chemotherapy; however, results are often poor and the median survival is approximately 6 months [5–7]. Improved therapy is urgently needed.

Several studies have demonstrated that hypoxia or hypoxia-inducible factors (HIFs), the primary mediators of the cellular response to hypoxia, play important roles in the biology of KSHV and KSHV-induced tumors. KS and PEL both tend to develop preferentially in anatomic locations with relative hypoxia (the feet and effusions), suggesting that hypoxia contributes to their oncogenesis [8–10]. The cellular response to hypoxia is primarily mediated by HIFs. There are two main isoforms of HIF, HIF-1 and HIF-2 [11]. Each is a heterodimer of two chains, α and β. Under normoxic conditions, HIF-1α and HIF-2α are constitutively expressed but are then rapidly ubiquitinated and destroyed [12]. Hypoxia leads to accumulation of the HIFα chain, which translocates to the nucleus and forms a dimer with HIF-1β. The HIF dimer then binds to hypoxia response element (HRE) sequences in the promoter region of hypoxia-sensitive genes and activates them [13]. HIF-1α and HIF-2α vary in their cell distribution; HIF-1α is expressed on a substantially wider range of cells than HIF-2α and is believed to play a more important role in the response to hypoxia [14, 15]. While most HIF-responsive genes are activated by both HIF-1α and HIF-2α, some are relatively specific for one or the other [14–16]. In particular, several proteins that catalyze steps in glycolysis are predominantly activated by HIF-1α [16, 17]. HIFs can also be elevated in an oxygen-independent manner, primarily through increased protein synthesis caused by the activation of AKT/PI3K/mTOR and MAPK pathways [13, 18], by certain pro-survival cytokines, by growth factors, and by other signaling molecules [19–23].

Like other herpesviruses, KSHV has two phases, latency, in which only a few genes are expressed, and lytic replication, in which all genes are expressed and progeny virions are produced. Hypoxia and HIFs have been shown to activate KSHV lytic replication [10]. HIF-1α can directly activate the expression of a variety of KSHV genes including KSHV replication and transcription activator (RTA), which induces the cycle of lytic replication [9]. RTA activation by HIF-1α is enhanced by its association with KSHV latency-associated nuclear antigen (LANA) [24]. In addition, HIF-1α has been shown to directly activate LANA [25], and the ORF34-37 cluster of lytic genes [26]. At the same time, KSHV infection has been shown to increase the levels and activity of HIF-1α [27]. KSHV infection upregulates many of the same genes upregulated by hypoxia and also leads to enhancement of HIF-1α -mediated processes such as aerobic glycolysis [28, 29]. Furthermore, PEL-derived cells have increased HIF-1α levels compared to non PEL B cell tumor-derived cells [30]. Several KSHV genes have been reported to increase HIF-1α levels and/or activity. In particular, LANA leads to stabilization and nuclear accumulation of HIF-1α under normoxia [30, 31]. Also, KSHV-encoded G protein-coupled receptor (vGPCR), encoded by ORF74, increases HIF-1α transcriptional activity through ERK1/2 and MAPK pathways [32]; KSHV interferon regulatory factor 3 (vIRF3) directly binds to HIF-1α, blocking its normoxic degradation and leading to nuclear accumulation and increased activity [33]; and KSHV-encoded miRNAs can cause an upregulation of HIF-1α protein levels [34, 35]. Thus, KSHV infection can potentially lead to a positive feedback loop in which KSHV enhances HIF-1 levels and activity; the increased HIF-1 can in turn enhance KSHV gene expression. These features of KSHV suggest that interference with HIF-1α might be a therapeutic strategy in PEL and other KSHV-induced tumors.

To determine if HIF-1α can be a potential therapeutic target for KSHV-induced malignancies, it is important to understand the overall effect of HIF-1 suppression in the context of a naturally infected tumor cell model. Since cell lines derived from PEL are the only naturally infected tumor cells currently available, we sought to explore the potential for anti-HIF approaches in PEL. We observed that knocking down HIF-1α expression in two PEL cell lines using a lentivirus expressing shRNA to HIF-1α resulted in substantial effects on several important aspects of PEL biology including reduced cell growth, dysregulation of cellular metabolism, and decrease in the expression of various lytic and latent KSHV genes. These effects were observed not only under hypoxic conditions, but also under normoxia. In addition, we observed that a small molecule inhibitor of HIF-1α impaired the growth and proliferation of PEL cells, suggesting that HIF-1α plays a critical role in KSHV biology and PEL pathogenesis and that it might be an attractive target for the design of new therapies for KSHV-associated diseases.

## Results

### Generation of PELs with stable knockdown of HIF-1

**Fig 1:**
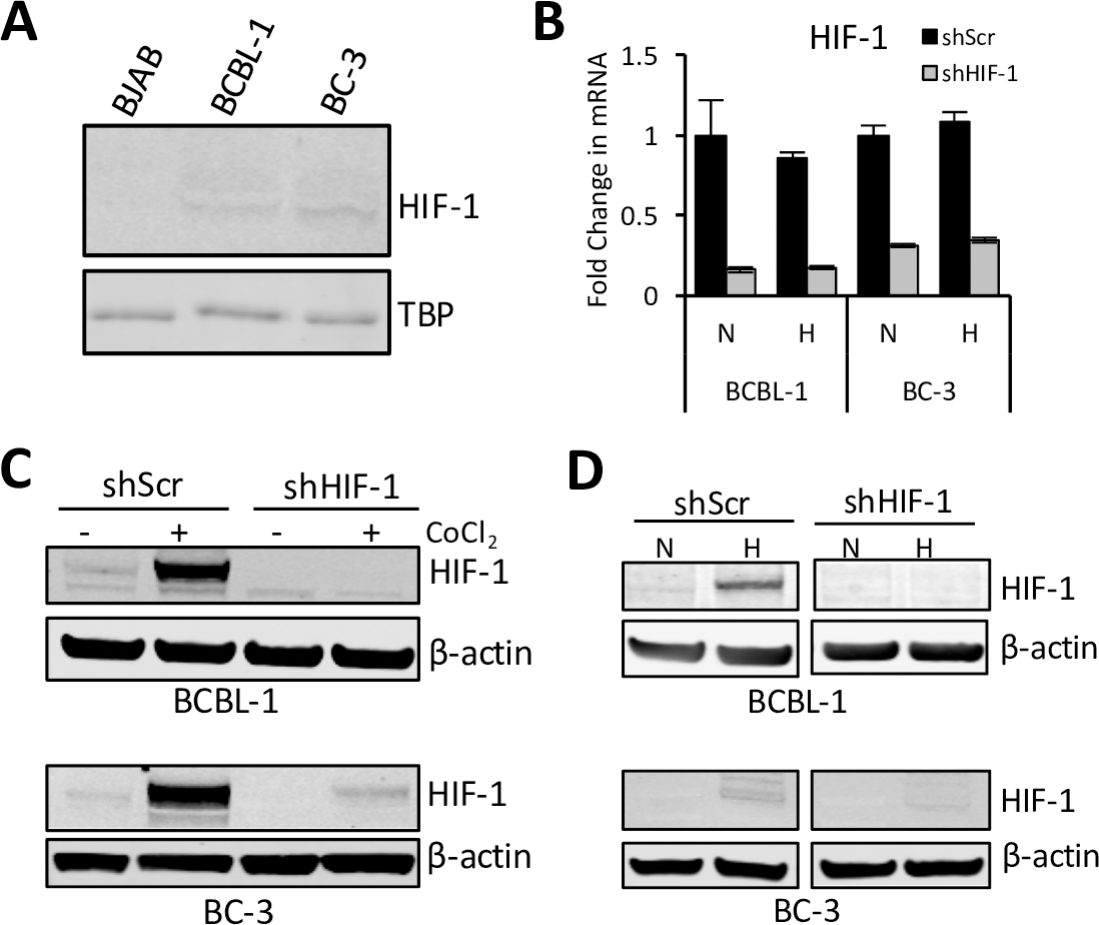
Generation of BCBL-1 and BC-3 cells with stable HIF-1α knockdown. **(A)** Protein levels of HIF-1α in nuclear extracts of BJAB, BCBL-1, and BC-3 cells measured by Western blot analysis after 24 hours in normoxia. Tata-binding protein (TBP) is used as a loading control. BCBL-1 and BC-3 cells were transduced with lentivirus encoding shRNA to HIF-1α or Scrambled (Scr) RNA and stable cell lines were generated with puromycin selection. Total RNA and nuclear protein extracts were extracted from the cells to confirm the status of the knockdown. (**B**) mRNA levels of HIF-1α measured by RT-qPCR after 48 hours in normoxia(N) or hypoxia(H). mRNA levels are normalized to that of 18S ribosomal RNA and are expressed as fold change relative to cells containing shScr under normoxia. (**C and D**) Protein levels of HIF-1α measured by Western blot analysis of nuclear extracts after 24 hours in culture. β-actin is shown as a loading control. (**C**) Normoxic levels of HIF-1α levels in the absence or presence of 50μM cobalt chloride (CoCl_2_), a hypoxia mimic that prevents oxygen-induced degradation of HIF-1α. (**D**) HIF-1α levels under normoxia or hypoxia.

We first determined the HIF-1α protein levels in PEL lines BCBL-1 and BC-3 and a virus-negative Burkitt’s lymphoma (BL) line BJAB. As observed in Fig 1A, PELs have detectable levels of HIF-1α under normoxia, unlike BJAB. Since HIF-1α is rapidly degraded in the presence of oxygen [36, 37], the detection of HIF-1α protein in normoxic PELs, albeit at low levels, is consistent with previous observations that HIF-1α protein’s normoxic degradation is prevented in PELs by various KSHV genes [30, 31, 33]. Next, to explore the role of increased HIF-1α in PELs, we infected BCBL-1 and BC-3 lines with a lentivirus encoding shRNA to HIF-1α to generate stable lines with suppressed HIF-1α (shHIF-1). BCBL-1 and BC-3 lines infected in parallel with a scrambled shRNA (shScr) were used as controls. After approximately 2 months of selection, the mRNA level of HIF-1α was reduced to approximately 20% and 30% in the BCBL-1 and BC-3 shHIF-1 cells respectively as compared to shScr cells (Fig 1B). As expected, there was no change in HIF-1α mRNA level when cells were placed under hypoxic conditions. We also measured the protein levels of HIF-1α under normoxia, in the absence or presence of CoCl_2_, a hypoxia mimic that prevents degradation of HIF-1α in the presence of oxygen. In shScr cells, HIF-1α was detectable at low levels in the absence of CoCl_2_, and its levels increased significantly in the presence of CoCl_2_ (Fig 1C). shHIF-1 cells, on the other hand, had undetectable levels of HIF-1α in the absence of CoCl_2,_ and exposure to CoCl_2_ either did not increase its level (BCBL-1) or increased it only slightly (BC-3) compared to shScr cells (Fig 1C). Similar results were obtained upon exposing the cells to hypoxia (Fig 1D), confirming that HIF-1α expression is reduced in shHIF-1 cells compared to shScr cells.

### HIF-1α knockdown results in reduced glycolysis and lipid biogenesis in PELs

**Fig 2:**
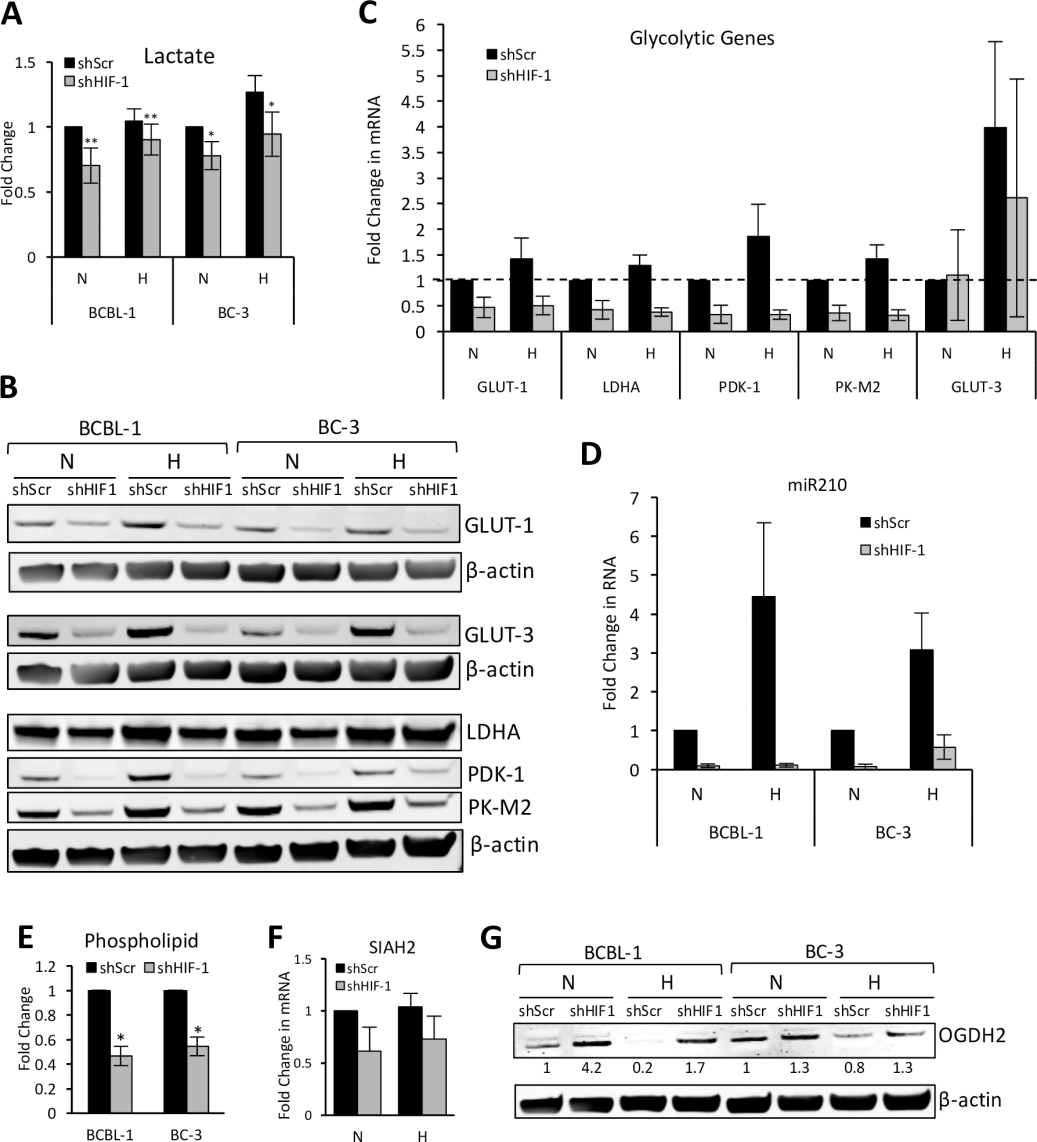
Effect of HIF-1α knockdown on glycolysis and lipid biogenesis. (**A)** Levels of extracellular lactate produced after 24 hours in normoxia (N) or hypoxia (H), expressed as fold change relative to cells containing shScr under normoxia. (**B)** Protein levels of HIF-1α regulated glycolytic genes after 48 hours in normoxia or hypoxia as assessed by Western blot. β-actin is shown as a loading control. (**C)** mRNA levels of HIF-1α regulated glycolytic genes measured by RT-qPCR after 48 hours in N or H. mRNA levels are normalized to that of 18S ribosomal RNA and are expressed as fold change relative to cells containing shScr under N. (**D**) Levels of miR210 measured by taqman RT-qPCR assay. RNA levels are normalized to that of internal control RNU43 and expressed as fold change relative to shScr cells in normoxia. (**E**) Levels of phospholipids produced in shHIF-1 cells relative to shScr cells in normoxia. (**F)** mRNA levels of SIAH2 in BCBL-1 cells normalized to 18S and expressed as fold change relative to shScr cells under N. (**G)** Protein levels of OGDH2 after 48 hours in N or H. Numbers below the blot for OGDH2 represent protein levels normalized to β-actin and expressed as fold change relative to shScr cells in N. Western blots were done on lysates from three independent experiments and representative blots are shown. Error bars represent standard deviations from at least 3 independent experiments. Statistically significant differences between shScr and shHIF-1 cells are indicated. *P ≤0.05, **P ≤ 0.01 (2-tailed t-test, paired).

Previous studies have shown that PELs have increased aerobic glycolysis relative to primary B cells, and that glycolytic inhibitors can reduce the growth and survival of BCBL-1 cells [38]. Consistent with these findings, we observed a dose-dependent decrease in proliferation and increase in cell death upon treatment of both BCBL-1 and BC-3 cells with the glycolysis inhibitor 2-DG (S1 Fig). Since HIF-1 regulates the expression of several genes in the glycolytic pathway, we hypothesized that the knockdown of HIF-1α in PEL cells might reduce glycolysis in PELs. shHIF-1 cells produced significantly lower levels of lactate relative to shScr cells under normoxia and hypoxia (Fig 2A) indicating that glycolysis is reduced in PELs upon HIF-1α knockdown. We then measured the levels of putative HIF-1 regulated glycolytic enzymes to understand the mechanism of reduced glycolysis in shHIF-1 cells (Fig 2B). HIF-1 knockdown led to decrease in the levels of glucose transporters GLUT-1 and GLUT-3; LDHA (lactate dehydrogenase A), which mediates the conversion of pyruvate into lactate; PDK-1 (pyruvate dehydrogenase kinase 1), which prevents oxidative decarboxylation of pyruvate, thereby preventing pyruvate from being used in oxidative phosphorylation; and PK-M2 (M2 isoform of pyruvate kinase), which is a HIF-1 responsive gene shown to be necessary for KSHV-induced increased glycolysis in endothelial cells [39] (Fig 2B). However, the level of another putative HIF-1 responsive gene, HK-2 (hexokinase 2), which phosphorylates glucose as the first step in glucose metabolism, was not consistently altered by HIF-1α knockdown (S2 Fig). We also measured the mRNA levels of these genes and found that all the genes measured, except GLUT-3, were also reduced at the RNA level, suggesting that their decrease is due to reduced transcription upon HIF-1α knockdown (Fig 2C).

Next, we determined the levels of a cellular miRNA miR210, which has been shown to be a HIF-1α-dependent gene in a variety of cell types [40–42]. miR210 has been shown to contribute to oncogenesis and have a number of other effects in cells [43–45]. Two of the validated targets of miR210 are iron-sulfur cluster scaffold proteins, ISCU1 and ISCU2, which are components of the mitochondrial electron transport chain [46]. miR-210 mediated downregulation of ISCU1/2 has been shown to shift cellular metabolism from oxidative phosphorylation to increased glycolysis [47, 48]. shHIF-1 cells showed an approximately 90% reduction in miR210 level (Fig 2D), not only confirming that the low baseline level of HIF-1α protein in normoxia is functional, but also suggesting that increased ISCU1/2 due to down-regulated miR210 might be yet another mechanism for reduced glycolysis in shHIF-1 cells. Together, these observations suggest that the reduced glycolysis in shHIF-1 cells is mediated at least in part due to reduced expression of some key HIF-1-responsive glycolytic genes upon HIF-1α knockdown.

A related feature of tumor cell metabolism is increased lipid biogenesis, which is responsible for most of the lipids required for their proliferation [49]. PELs also have increased lipid synthesis and a number of phospholipids are amongst the most upregulated lipid components in PELs compared to primary B cells [50]. Since HIF-1 can regulate the level of FasN, an important enzyme complex required for lipid synthesis that has been shown to be upregulated in PELs [50], we wanted to determine if lipid synthesis is affected by HIF-1α knockdown. We found that shHIF-1 PEL cells have approximately 50% decrease in phospholipids compared to control shScr PEL cells (Fig 2E). To understand the mechanism of reduced phospholipids in shHIF-1 cells, we determined the protein levels of putative HIF-1-responsive genes in the lipid synthesis pathway. Surprisingly, the levels of two of the key proteins in the pathway, FasN and Lipin 1, were both unchanged in shHIF-1 cells (S2 Fig) suggesting that changes in the levels or activity of other HIF-1 responsive enzymes may be primarily responsible for the changes in phospholipids.

Lipid biosynthesis can also be regulated by the 48kDa splice variant of oxoglutarate dehydrogenase (OGDH2), a subunit of αketoglutarate dehydrogenase (αKGDH). αKGDH is a mitochondrial enzyme complex that catalyzes the oxidation of αketoglutarate (αKG) into succinate for use in the tricarboxylic acid (TCA) cycle. A decrease in the level of OGDH2, causes reduced entry of αKG into the TCA cycle and favors its incorporation into lipid synthesis [51]. HIF-1 has been shown to upregulate the expression of an E3 ubiquitin ligase, SIAH2, that targets OGDH2 for degradation [51]. We observed a reduction in SIAH2 RNA level upon HIF-1α knockdown (Fig 2F). Consistent with this observation, the level of OGDH2 was increased in shHIF-1 cells compared to shScr cells in both cell lines (Fig 2G), although the increase was not as pronounced in BC-3 cells likely due to incomplete HIF-1α knockdown in these cells (Fig 1C and 1D). Together, these results indicate that the reduced lipid production in shHIF-1 cells is most likely due to increased αKGDH activity.

### HIF-1α is required for both normoxic basal lytic replication and hypoxia-induced lytic replication of KSHV

**Fig 3:**
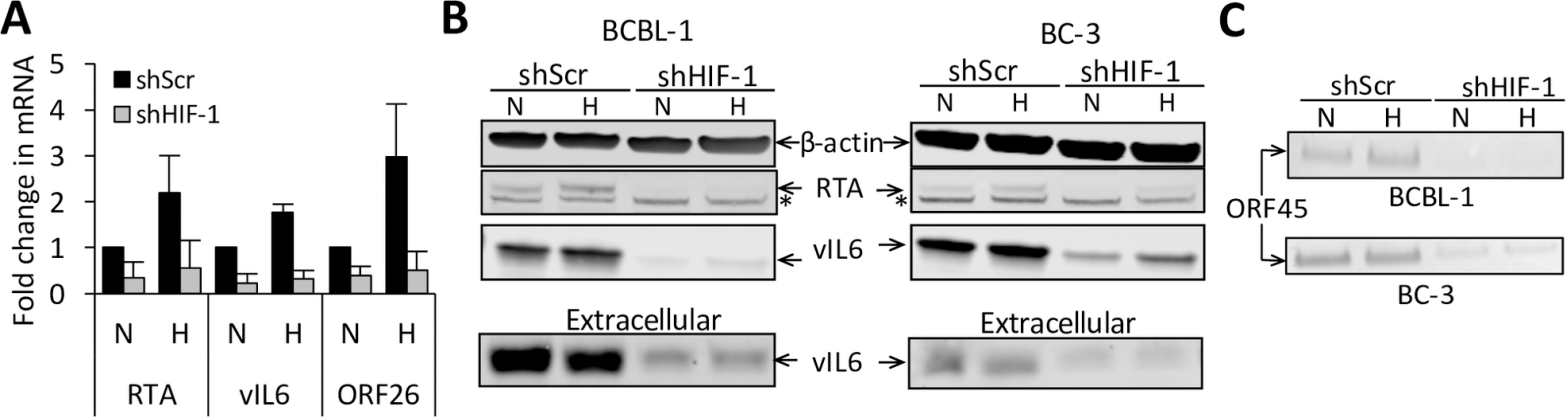
HIF-1α knockdown leads to reduced lytic replication of KSHV. **(A)** mRNA levels of RTA, vIL-6, and ORF26 (a late lytic gene) of KSHV in BCBL-1 cells measured by RT-qPCR. Error bars represent standard deviation from at least 3 experiments. (**B)** Western blot showing protein levels of intracellular RTA, vIL6, and β-actin in the cell lysates as well as secreted vIL6 in the supernatant after 48 hours in normoxia (N) or hypoxia (H). (**C)** Protein levels of ORF45 in concentrated virus particles released in the supernatants after 72 hours. *Unspecific band. Western blots were done on lysates from three independent experiments and representative blots are shown.

Hypoxia induces lytic reactivation of KSHV [10], and HIF-1 has been shown to directly upregulate the level of KSHV lytic switch protein RTA, especially in the presence of LANA [9, 24]. This suggested that knockdown of HIF-1α might impair KSHV lytic activation in PEL cells exposed to hypoxia. To assess this, we measured the mRNA levels of three KSHV lytic genes, RTA (immediate-early), vIL6 (early), and ORF26 (late) in shHIF-1 PEL cells (Fig 3A). We found that the mRNA of all three genes increased with hypoxia but decreased substantially upon HIF-1α knockdown, both in normoxia and hypoxia (Fig 3A). We also measured the protein levels of intracellular RTA and vIL6 in cell lysates as well as secreted vIL6 levels in the supernatants using Western blotting, and found that the changes in their protein levels correlated with changes in their mRNA levels; shHIF-1 decreased their levels under hypoxia and also suppressed their basal levels under normoxia (Fig 3B). We then examined the effects of HIF-1α knockdown on virus production. Virus particles from the supernatants were collected after 72 hours in normoxia or hypoxia, concentrated by ultracentrifugation, lysed, and Western blotting was performed for ORF45, a lytic protein included within the tegument of KSHV virus particle. As has been previously described, hypoxia induced a small increase in ORF45 production, however, shHIF-1 cells had a decrease in ORF45 production in both normoxia and hypoxia to levels substantially below the basal level seen in normoxic shScr cells (Fig 3C). These observations indicate that HIF-1 plays an important role in lytic reactivation of KSHV in normoxia as well as hypoxia.

### HIF-1α is important for the expression of latent genes of KSHV

**Fig 4:**
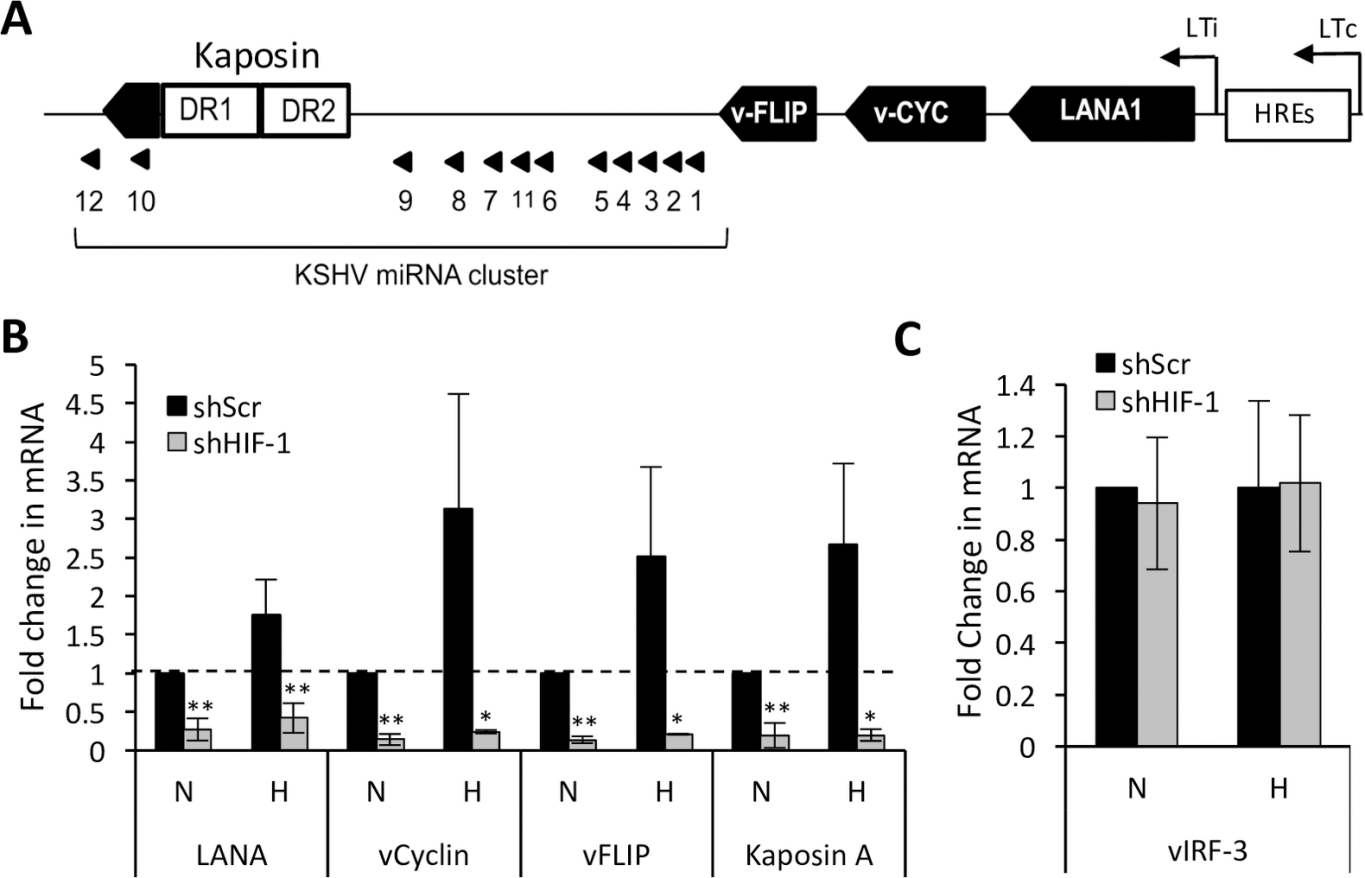
Effect of HIF-1α knockdown on the expression of KSHV latent genes in PELs. (**A**) Schematic of multicistronic KSHV latency locus showing the location of latent genes and hypoxia-response elements (HREs) in the promoter region. (**B**) mRNA levels of latent genes in BCBL-1 cells within and (**C**) outside of the latency locus as measured by RT-qPCR and normalized to that of 18S RNA. RNA levels for each gene are expressed as fold change relative to shScr cells under normoxia (N). Error bars represent standard deviations from at least 3 independent experiments. Statistically significant differences between shScr and shHIF-1 cells are indicated. *P ≤0.05, **P ≤ 0.01 (2-tailed t-test).

The latency locus of KSHV contains several HREs in the promoter region (Fig 4A). HIFs have been shown to bind to those HREs, and hypoxia can upregulate LANA in PELs [25]. Therefore, we wanted to test if the suppression of HIF-1α leads to a change in LANA levels. We found a substantial decrease in the mRNA level of LANA in shHIF-1 cells not only under hypoxia, but also under normoxia (Fig 4B). We verified that this decrease in normoxia was associated with a decrease in its protein level by performing Western blot analysis of nuclear lysates of BCBL-1 cells (S3 Fig). Since the promoter region upstream of LANA can also upregulate ORFs containing latent genes vCyclin, vFLIP, and Kaposin located within the latency locus through alternate splicing, we examined the mRNA levels of these genes and found that the levels of vCyclin, vFLIP, and Kaposin A are all increased by hypoxia and substantially decreased upon HIF-1α suppression (Fig 4B). By contrast the level of vIRF3, another latent gene in PEL, that is encoded outside of the main latency locus did not change either with hypoxia or upon HIF-1α knockdown (Fig 4C) suggesting that binding of HIF-1α to the HREs found in the promoter region of the latency locus is the primary mechanism by which HIF-1α regulates the expression of KSHV latent genes.

**Fig 5:**
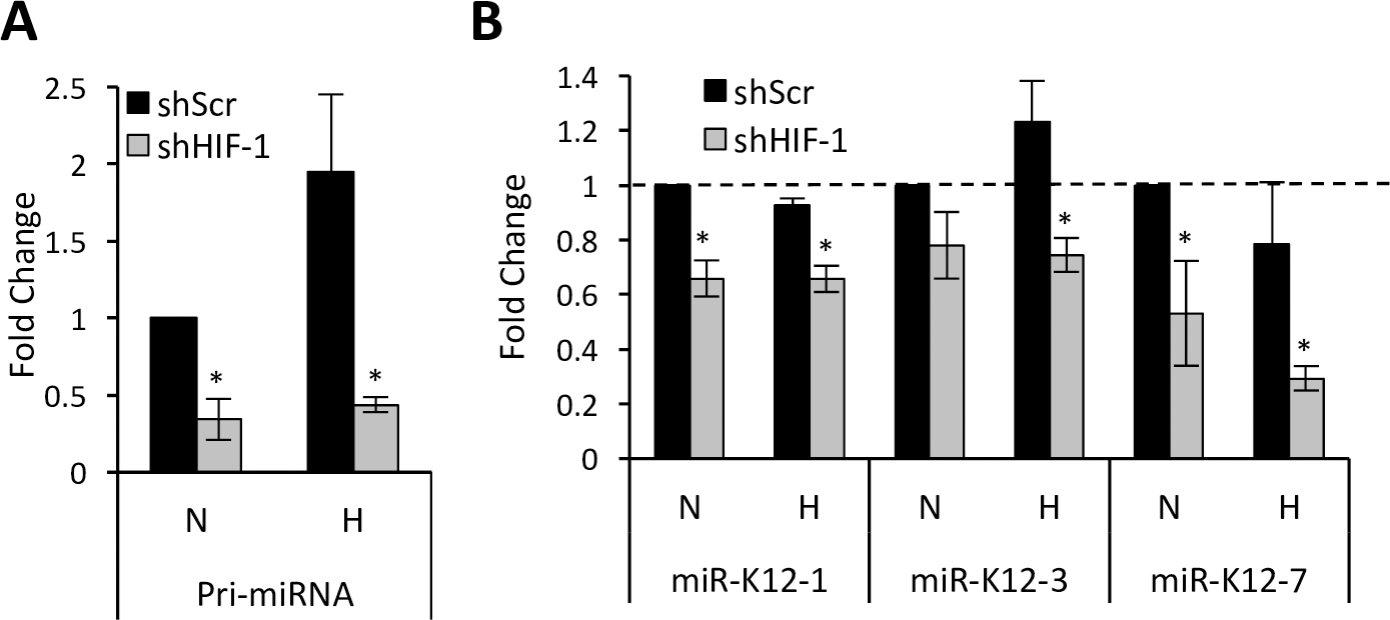
Effect of HIF-1α knockdown on the expression of KSHV miRNAs. **(A)** Level of primary miRNA transcript as measured by RT-qPCR and normalized to 18S mRNA. **(B)** Levels of mature miRNAs measured using taqman assays and normalized to that of RNU43 miRNA. Error bars represent standard deviations from at least 3 independent experiments. Statistically significant differences between shScr and shHIF-1 cells are indicated. *P ≤0.05 (2-tailed t-test).

The latency locus also encodes miRNAs that are expressed during latency, and we assessed whether the miRNAs were similarly suppressed in shHIF-1 cells. We first performed RT-qPCR using primers directed to the intergenic region of the latency locus and found that the primary miRNA (pri-miRNA) transcript, which produces mature miRNAs after downstream processing, is increased during hypoxia and decreased upon HIF-1α knockdown (Fig 5A). We then measured the levels of mature miRNAs and found that they were unchanged by hypoxia (Fig 5B and S4 Fig). This finding is consistent with previous observations on the effect of hypoxia on KSHV miRNAs in SLK/SLKK cell model [28]. However, the levels of each of the 3 mature miRNAs tested (miR-K12-1, miR-K12-3, and miR-K12-7) were significantly reduced by HIF-1α suppression under conditions of both normoxia and hypoxia (Fig 5B). This observation suggests that while the basal HIF-1 activity in PEL cells is important for the maintenance of the mature miRNA levels and hypoxia can increase pri-miRNA, it does not increase mature miRNA levels, perhaps because an increase in primary miRNA levels is counterbalanced by reduced miRNA processing under conditions of hypoxia.

### HIF-1α is important for the growth of PEL cells

**Fig 6:**
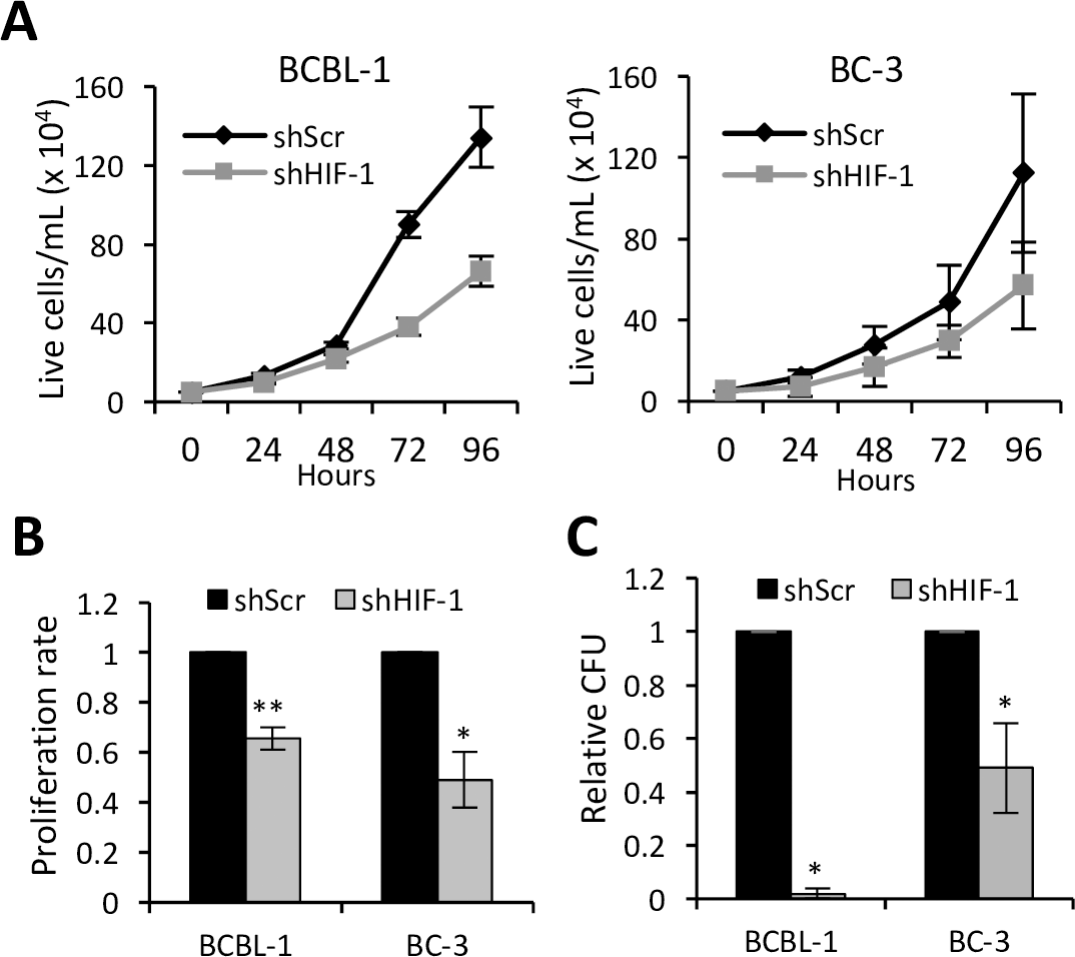
Effect of HIF-1α suppression on growth of PELs. **(A)** Growth rate of shScr or shHIF-1 BCBL-1 and BC-3 cells measured by counting live cells every 24 hours in normoxia. (**B)** Proliferation rate of shScr or shHIF-1 cells measured by MTS assay at 72 hours in normoxia using Promega’s CellTiter 96 Aqueous One Solution assay, which measures the amount of NADH or NADPH produced by metabolically active cells. (**C)** Colony forming efficiency of shHIF-1 cells relative to shScr cells. Error bars represent standard deviations from at least 3 independent experiments. Results shown in B and C are fold changes compared to shScr cells. Statistically significant differences between shScr and shHIF-1 cells are indicated. *P ≤0.05, **P ≤ 0.01.

Since we found that HIF-1α knockdown affects not only glycolysis and lipid production, which are important for the PEL cell growth, but also the expression of various KSHV genes known to promote the growth and survival of PELs, we wondered if HIF-1α expression was important in maintaining the growth of PEL cells. In normoxia, shHIF-1 cells grew substantially slower than the shScr control cells (Fig 6A). This difference in growth could be attributed to slower proliferation rates of shHIF-1 cells, which were approximately 0.6 and 0.5 fold compared to that of shScr BCBL-1 and BC-3 cells respectively as measured using a proliferation-specific MTS assay (Fig 6B). We also determined the ability of these cells to form colonies at limiting dilution conditions and found that the shHIF-1 BCBL-1 and BC-3 cells formed colonies with approximately 2% and 50% the efficiency of the respective shScr cells (Fig 6C).

### HIF-1α inhibitor PX-478 is effective against PELs

**Fig 7:**
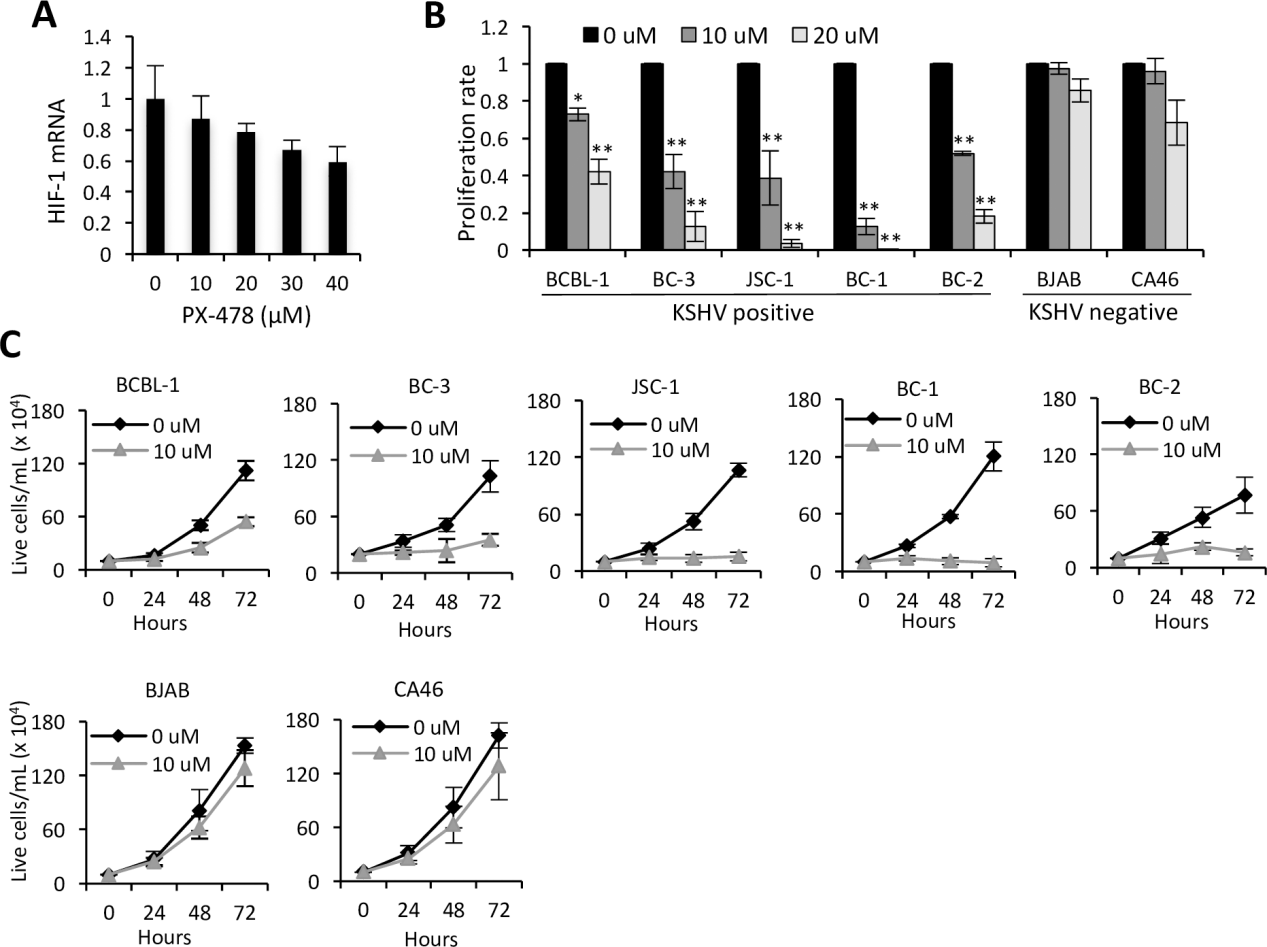
Effects of HIF-1α inhibitor PX-478 on PELs. (**A**) HIF-1α mRNA levels in BCBL-1 cells 24 hours after treatment with various concentrations of PX-478, normalized to 18S internal control and expressed as fold changes compared to no PX-478 control cells. (**B)** Proliferation rate of PEL cell lines BCBL-1, BC-3, JSC-1, BC-1, and BC-2 and Burkitt’s lymphoma (BL) cell lines BJAB and CA46 measured using the MTS assay 72 hours after treatment with indicated concentrations of PX-478, expressed as fold changes compared to no PX-478 control cells. (**C)** Growth rates of the PEL and BL cells treated with 0 or 10 μM PX-478. Error bars represent standard deviations from at least 3 independent experiments. Statistically significant differences between untreated and inhibitor-treated cells are indicated. *P ≤0.05, **P ≤ 0.01.

To test whether the suppression of HIF-1α can be explored as a treatment strategy for PEL, we utilized PX-478, a small molecule that has been shown to potently inhibit the expression of HIF-1α [52] in a variety of tumor models [53] and mice [54]. PX-478 suppresses HIF-1α at several levels, including an effect on HIF-1 mRNA. First, we determined that PX-478 in fact induced a dose-dependent decrease in HIF-1α mRNA in BCBL-1 cells (Fig 7A). Next, we examined the effect of PX-478 on the proliferation rates of various PEL cell lines as well as virus-negative BL cell lines, BJAB and CA46. We found that the proliferation rates of all the PEL cell lines tested (KSHV-positive but EBV-negative lines BCBL-1 and BC-3, KSHV and EBV dual positive lines JSC-1, BC-1, and BC-2) were significantly reduced when treated with 10μM or 20μM PX-478 (Fig 7B). By contrast, the proliferation rates of the two BL lines were not affected by 10μM, and minimally affected by 20μM PX-478 (Fig 7B). Consistent with this observation, the growth of PEL lines but not the BL lines was slowed when treated with 10μM PX-478 (Fig 7C). To further test the increased sensitivity of KSHV-infected cells to HIF-1 inhibitor, we performed growth assays of Vero cells with or without latent KSHV. PX-478 treatment reduced the proliferation rates and growth of VERO-K cells substantially more than that of VERO cells (S5 Fig). Combined, these results suggest that HIF-1α suppression preferentially and substantially inhibits the growth of KSHV-positive cells and that this effect can be achieved using a small-molecule inhibitor of HIF-1α.

## Discussion

In this study, we demonstrate that HIF-1α plays a key role in the biology of KSHV infection. We show that PEL cell lines have detectable levels of HIF-1α, even under normoxic conditions and that HIF-1α in these PEL lines is important for the maintenance of cell growth, upregulation of glycolysis, and expression of several KSHV genes that are important for oncogenicity of KSHV. Thus, PEL lines have a positive feedback loop in which KSHV infection upregulates HIF-1α, which in turn contributes to the growth and survival of these cells. We demonstrate that knockdown of HIF-1α in these lines can interfere with these processes. Finally, we show that a small molecule inhibitor of HIF-1α, PX-478, can impair the growth of KSHV-positive cells at concentrations that have little or no effect on KSHV-negative cells, suggesting that suppression of HIF-1 may be worth exploring as a therapy for PEL.

While HIF-1α protein is normally undetectable under normoxia due to its rapid oxygen-dependent degradation [36, 37], we observed low but detectable levels of HIF-1α protein even in normoxic PEL cells. This observation confirms previous findings that KSHV infection can enhance not only HIF-1 activity but also its levels through increased transcription or stabilization; these effects are mediated by several KSHV genes including LANA (ORF73), K1, vIRF3, vGPCR (ORF74), and KSHV-encoded miRNAs [24, 27, 30, 32–34, 39, 55–58]. Our work further extends these observations and demonstrates that even the low levels of HIF-1α present in normoxic PEL cells functions to affect HIF-1 target genes, and maintain the growth of these cells, their metabolic features, and the expression of key KSHV genes. As noted above, PEL develops in a hypoxia environment (pleural and other effusions), and in this setting HIF levels are even higher and there would be even more pronounced activation of HIF-1-mediated processes.

KSHV infection has been shown to increase the glycolytic genes, GLUT-1 and GLUT-3, as well as glycolysis in several endothelial cells such as TIME, hDMVECs, HUVEC, and LEC [29, 34, 39]. These effects are somewhat variable, however, as KSHV infection was found in a recent report to decrease glycolysis in rat primary mesenchymal precursor cells (MM) and BJAB Burkitt’s lymphoma cells [59]. Interestingly, both MM and BJAB cells have highly elevated basal level of aerobic glycolysis [59, 60]. Therefore, while the exact reason for the contradictory effects of KSHV on glycolysis is not clear, it seems likely that at least in cells with relatively low basal glycolysis, KSHV enhances glycolysis at least in part through an increase in HIF-1 to generate an optimal level of aerobic glycolysis needed for growth and transformation of cells post-infection. In this regard, PELs have been shown to have upregulated aerobic glycolysis as well as lipid synthesis compared to primary B cells [38]. Here, we show that the relatively high level of aerobic glycolysis in PEL cells is at least in part maintained by enhanced HIF-1 activity. In particular, we demonstrate that HIF-1α knockdown leads to a downregulation of aerobic glycolysis as shown by a decrease in extracellular lactate levels. We also show that this decrease is primarily due to reduced transcription of some key HIF-1-responsive glycolytic enzymes such as GLUT-1, PK-M2, LDHA, and PDK-1, as well as a cellular miRNA miR210. Interestingly, the level of GLUT-3 protein but not RNA was reduced upon HIF-1α knockdown, suggesting a post-transcriptional mechanism of GLUT-3 regulation by HIF-1. It is noteworthy that while statistically significant, the decrease in lactate level upon HIF-1α knockdown is minimal suggesting that the low/residual level of glycolytic genes expressed in shHIF-1 cells might be sufficient for some level of aerobic glycolysis. Alternatively, it is also possible that HIF-1 is only partially responsible for the increased aerobic glycolysis in PELs. cMyc, which can regulate glycolysis as well as the expression of some of the same glycolytic genes as HIF-1 [61], is expressed in PELs and unchanged by HIF-1α knockdown (S6 Fig), suggesting that the residual aerobic glycolysis observed in shHIF-1 cells is potentially due to cMyc activity.

We also show that HIF-1α knockdown in PELs results in reduced lipid synthesis. We found that this decrease is likely not due to changes in Lipin 1 or FasN, which was shown to be necessary for increased lipid synthesis in PELs [38]. Instead our data suggests that a decrease in citrate due to increased levels of OGDH2, which is degraded by a HIF-1-responsive ubiquitin ligase SIAH2 [51], is responsible for decreased lipid synthesis observed upon HIF-1α knockdown. This interpretation is supported by our observation that SIAH2 RNA level is reduced while OGDH2 protein level is increased in shHIF-1 cells.

HIF-1 can induce KSHV lytic replication through activation of RTA, and can also activate certain KSHV genes directly [9, 10, 26, 31]. While most PEL cells have KSHV in a latent state, there is some evidence that there is limited expression of lytic genes and that certain of these genes, such as vGPCR (ORF74) or vIL-6 (which is also expressed under latency in PEL, albeit at lower levels) are important in the pathogenesis of KSHV-related tumors [56, 62]. These observations were thought to be important only in the context of hypoxia, but in the current study, we show that HIF-1 is important for the expression of KSHV lytic genes in PEL cell lines even in normoxia. In particular, we demonstrate that the expression of RTA, vIL-6, and the late-lytic gene ORF26, as well as production of virus were all decreased upon HIF-1α knockdown. While the stress and increased HIF-1α levels associated with hypoxia can increase KSHV lytic reactivation, our data shows that the basal level of HIF-1α in PELs is sufficient and necessary for the basal KSHV lytic reactivation that occurs in these cells under normoxia.

We also show that even under conditions of normoxia, the HIF-1α present in PEL cells is important in maintaining the production of KSHV latent genes. Most of the latent KSHV genes are organized in a cluster called the latency locus, which includes latent proteins LANA, vCyclin, vFLIP, and kaposins. These genes have all been shown to play roles in the oncogenesis by KSHV through a variety of mechanisms including inhibition of apoptosis, activation of NF-κB, and induction of cell proliferation [63, 64]. Our group previously showed that hypoxia and HIFs could activate LANA by the binding of HIFs to HREs in the LANA promoter region [25]. Here, we extend these observations and provide evidence that HIF-1 maintains levels of not only LANA but also vCyclin, vFLIP, and kaposin. In particular, we show that HIF-1α knockdown leads to an overall downregulation in the expression of these genes regardless of oxygen conditions. Interestingly, while we previously observed little change in the levels of kaposin protein when JSC-1 or BC-3 PEL cells were exposed to hypoxia [25], here we observed a substantial decrease in kaposin A mRNA under both normoxia and hypoxia when HIF-1α was knocked down. The reason for this discrepancy is not clear. However, it is noteworthy that another latent gene in PELs, vIRF3, which uses a different promoter that does not contain HREs was not affected by either hypoxia or HIF-1α knockdown in the current study. KSHV encodes several miRNAs in the latency region that play important roles in modulating the expression of host cell genes. In this study, we found that the primary miRNA transcript of KSHV is upregulated by hypoxia and suppressed by HIF-1α knockdown. We also show that while the levels of mature miRNAs are generally not enhanced by hypoxia, they are decreased by HIF-1α knockdown. The lack of response of the mature miRNA to hypoxia is consistent with a previous study in the SLK cell model system [28]. Although the exact reason for this discrepant observation is not known, it is possible that hypoxia-induced decreases in miRNA processing contributes to the lack of hypoxia-induced upregulation of the mature miRNAs.

In addition to shHIF-1 downregulating cellular metabolic processes and several KSHV genes that promote oncogenesis, we observed that PEL cells with shHIF-1 had impaired growth and colony forming ability compared to those with shScr. This growth impairment was primarily due to a slower rate of proliferation. This observation is particularly noteworthy when considering that the cells expressing shHIF-1 were selected over the course of several months and thus potentially had time to develop compensatory adjustments. Similar effects on the growth of PELs were observed with the small molecule HIF-1α inhibitor, PX-478. PX-478 selectively impaired the growth of PELs at a substantially lower dose relative to EBV-negative BL cell lines BJAB and CA46. Of note, BL cells also have elevated aerobic glycolysis and in particular, BJAB cells have been shown to have higher aerobic glycolysis relative to PELs [59]. However, aerobic glycolysis in BL cells is predominantly mediated by cMyc, and EBV-negative BL cell lines including BJAB have very low/undetectable level of HIF-1α in normoxia [30, 65]. These observations help explain the increased sensitivity of PELs to HIF-1α inhibition relative to BL lines. Furthermore, reduced expression of several growth-promoting KSHV genes in addition to reduced glycolysis and lipid synthesis upon HIF-1α suppression might account for the increased sensitivity of PELs to growth impairment by HIF-1α inhibitor. Moreover, inhibition of miR-210 upon HIF-1 suppression may, through a variety of pathways, suppress the proliferation of PEL cells [43–45]. Consistent with these findings, we observed that PX-478 affects the growth of KSHV-infected Vero cells more than that of non-infected Vero cells, suggesting that KSHV-infection renders cells more sensitive to HIF-1α inhibition.

In summary, our data supports and extends previous findings that show an extensive network of interactions between KSHV and HIF-1. We show that targeting a single gene, HIF-1α, can have dramatic effects on the oncogenic metabolic signature of PELs, the expression of KSHV-encoded oncogenes, as well as their growth suggesting that PEL cells are particularly vulnerable to suppression of HIF-1. A number of small molecule inhibitors for HIFs are under development [52, 66–68], and the results here suggest that their use is worth exploring in PEL and potentially other KSHV-induced tumors. Further experiments exploring the use of these inhibitors, alone or in combination with other cytotoxic agents, in vitro as well as in vivo settings will be necessary to fully establish HIF-1α as a therapeutic target for KSHV-associated malignancies.

## Materials and methods

### Cell culture

PEL cell lines BC-3, BC-1, BC-2 and EBV-negative BL cell lines, BJAB and CA46, were obtained from ATCC, Manassas, VA. PEL cell line BCBL-1 was obtained from National Institutes of Health AIDS Research and Reagent Program, and JSC-1 was a kind gift from Dr. Richard F. Ambinder, Johns Hopkins University School of Medicine, Baltimore, MD. Vero cells (African green monkey kidney cells) were obtained from ATCC, and Vero cells latently infected with recombinant KSHV rKSHV.219[69] (Vero-K) were kindly provided by Dr. Jeffrey Vieira, University of Washington, Seattle, WA. The B cell lines were grown in RPMI 1640 medium (ThermoFisher Scientific, Waltham, MA) supplemented with 15% fetal-bovine serum (FBS) (ThermoFisher Scientific), 1% penicillin/streptomycin glutamine, and 20μM β-mercaptoethanol (Sigma-Aldrich, St. Louis, MO) (complete media) at 37°C with 5% CO_2_. Vero cells were grown in DMEM medium (ThermoFisher Scientific) supplemented with 10% fetal-bovine serum (FBS) (ThermoFisher Scientific), and 1% penicillin/streptomycin glutamine. Stable PEL cell lines with scrambled or HIF-1α knockdown (shScr and shHIF-1 respectively) were generated using lentivirus (ThermoFisher Scientific) encoding shRNA to a scrambled or HIF-1α RNA. shScr and shHIF-1 PEL cells and Vero-K cells were maintained in the presence of 10 μg/mL puromycin. Where indicated, normoxic and hypoxic cells were grown in an incubator with 21% or 1% O_2_ respectively. All the cell lines were grown in culture for a maximum of 20 passages, and HIF-1α knockdown status in the shScr and shHIF-1 cells was verified by Western blotting for HIF-1α after every thawing cycle. Chemicals used to treat the cells include the hypoxia mimic CoCl_2_ (Sigma-Aldrich), HIF-1 inhibitor PX-478 (S7612, Selleck Chemical, Houston, TX), and glycolysis inhibitor 2-Deoxy-D-glucose (2-DG) (8375, Sigma-Aldrich).

### Lactate assay

Cells were plated in normoxia or hypoxia at 3x10^5^ cells/mL of complete media. Supernatant was collected after 24 hours and deproteinised using a 10KD filter (EMD Millipore, Billerica, MA) by centrifuging for 30 minutes at 10,000 g at room temperature. L-lactate was quantified using an assay kit (MAK064, Sigma-Aldrich) using the manufacturer’s protocol.

### Lipid extraction and quantification

Cells were plated at 3 x 10^5^ cells per mL. 48 hours later, lipids were extracted using Chloroform-free lipid extraction kit (K216, Biovision, Milipitas, CA) according to the manufacturer’s protocol. Briefly, 10^6^ cells were resuspended with 50 μL phosphate-buffered saline (PBS). Lipid was extracted with 1 mL of lipid extraction buffer and dried overnight in a vacuum concentrator. Phospholipid content was quantified using phospholipid assay kit (MAK122, Sigma-Aldrich). Briefly, lipids dried overnight were resuspended in 100 μL of assay buffer for phospholipids, vortexed vigorously for 5 minutes, and quantified as per the manufacturer’s protocols.

### Western blot analysis

Cells were plated at 3 x 10^5^ cells per mL in normoxia or hypoxia for 48 hours. Nuclear and cytoplasmic extracts or whole cell lysates were prepared using NE-PER Nuclear Extraction kit or the M-PER mammalian protein extraction reagent (ThermoFisher Scientific), respectively, according to the manufacturer’s protocol. Protein concentrations were determined using the Pierce BCA Protein Assay Kit (ThermoFisher Scientific). Equal amounts of protein were subjected to LDS-PAGE using 4 to 12% NuPAGE Bis-Tris precast gels (ThermoFisher Scientific) and transferred to a nitrocellulose membrane using iBlot dry blotting system (ThermoFisher Scientific). The membrane was blocked either with 5% w/v nonfat dry milk (Santa Cruz Biotechnology Inc., Dallas, TX) in 1X TBST, made using 10X TBS buffer pH 7.4 (Quality Biological Inc., Gaithersburg, MD) and 0.05% Tween 20 (Promega, Madison, WI) for use with alkaline phosphatase (AP) colorimetric detection system or with Odyssey blocking buffer (Li-Cor BioSciences, Lincoln, NE) for use with the Licor fluorescent detection system. Blots were incubated with the primary antibodies listed in S1 Table for 1 hour at room temperature or overnight at 4°C. After 3 washes with TBST, the blots were then incubated for 1 hour with the appropriate alkaline phosphatase-conjugated secondary antibodies for visualization by Calf intestinal alkaline phosphatase (Promega) or with IRDye 800CW or 680LT secondary antibodies (Li-Cor Biosciences) conjugated with green or red fluorescent dyes for visualization using Odyssey imaging system (Li-Cor Biosciences). Western blots were analyzed using ImageStudio (Li-Cor Biosciences).

### Analysis of KSHV virus production by Western blot

Cells were plated at 2 x 10^5^ cells per mL in normoxia or hypoxia. After 72 hours, supernatant was collected, and processed for western blotting as described previously [70]. Equal volumes of the resuspended pellet containing concentrated virus were used for Western blot analysis of ORF45, a tegument protein contained in the virus particle, as described above.

### Real-time quantitative-PCR (RT-qPCR)

Cells were plated at 3 x 10^5^ cells per mL under normoxia or hypoxia and total RNA was extracted using *mir*Vana kit (ThermoFisher Scientific). cDNA synthesis for mRNA analysis by q-PCR was performed using High-Capacity cDNA Reverse Transcription kit (ThermoFisher Scientific) as per the manufacturer’s protocol on a T100 Thermal Cycler (Bio-Rad, Hercules, CA). The thermal conditions were 25°C for 10 minutes, 37°C for 2 hours, and 85°C for 5 minutes. qPCR was performed using *Power* SYBR Green PCR Master Mix (Applied Biosystems, Foster City, CA) on a StepOnePlus Real-Time PCR System (Applied Biosystems). q-PCR reaction setup included enzyme activation at 95°C for 10 mins and 40 cycles of amplification at 95°C for 15 sec and 60°C for 1 min followed by melting curve analysis according to the instrument standard instructions. mRNA expression was normalized to that of 18S endogenous control RNA and the quantification of relative mRNA expression was performed using ΔΔCt method. The primers used for q-PCR were either synthesized by Lofstrand Labs Limited, Gaithersburg, MD or purchased from Bio-Rad (Hercules, CA) as ready-made assays. The sequences of primers synthesized and the assay numbers of the ones purchased from Bio-Rad are listed in S2 and S3 Tables, respectively.

### Taqman assays for miRNAs

cDNA synthesis for miRNA analysis was performed on total RNA using TaqMan MicroRNA reverse transcription kit (ThermoFisher Scientific), and stem-loop qPCR on the cDNA templates was performed using Taqman Universal master mix II (ThermoFisher Scientific). Reverse transcription and q-PCR were performed using primers from TaqMan MicroRNA Assays (ThermoFisher Scientific) according to manufacturer’s instructions. The assay numbers are listed in S4 Table. miRNA expression was normalized to that of RNU43 endogenous control miRNA and the quantification of relative miRNA expression was performed using ΔΔCt method.

### Growth assays

Growth and viability assays were performed by plating the cells at 5-10x10^4^ cells/mL and counting the live and dead cells every 24 hours by trypan blue exclusion method to get growth curves. Proliferation rate was measured at 72 hours using CellTiter 96 Aqueous One Solution Cell Proliferation Assay (Promega) using the recommended protocol. Colony formation assay was performed similarly to that described previously [71, 72]. Briefly, cells were plated at 100, 10, or 1 per well of 96-well plates (1 plate/dilution) and grown for approximately 2–3 weeks. The numbers of wells without outgrowing colonies were counted and colony forming unit (CFU) was calculated using the Poisson distribution as: CFU = [(-ln[number of negative wells / total number of wells]) / number of cells per well] x 100%

### Statistical analysis

Statistical analysis was performed using two-tailed student’s t-test on experiments with at least 3 biological replicates. *P*-values less or equal to 0.05 were considered statistically significant.

## Supporting information

S1 Fig PEL cells are sensitive to inhibition of glycolysis. Cells were treated with various concentrations of glycolysis inhibitor 2-DG for 24 hours and rate of proliferation (**A**) and cell death (**B**) were measured using MTS assay and trypan blue exclusion method, respectively. Results are expressed as fold changes compared to no 2-DG control. Error bars represent standard deviations from at least 3 independent experiments.
(TIF)

S2 Fig Protein levels of metabolic genes unchanged by HIF-1α knockdown. Protein levels of HK-2, Lipin 1, and FasN were measured in shScr and shHIF-1 cells after 48 hours in normoxia (N) or hypoxia (H). β-actin is shown as a loading control.
(TIF)

S3 Fig LANA protein levels. Western blot of LANA in the nuclear extracts of BCBL-1 cells cultured for 48 hours. β-actin is shown as a loading control.
(TIF)

S4 Fig Effect of hypoxia on the levels of KSHV miRNAs in BCBL-1 cells. Levels of mature KSHV miRNAs were measured using taqman assays. The miRNA levels were normalized to that of RNU43 internal miRNA control and the results are expressed as fold change over normoxia.
(TIF)

S5 Fig Effects of HIF-1α inhibitor PX-478 on VERO and VERO-K cells. VERO and VERO-K cells were plated at 5x10^4^ cells per well of a 6-well plate. Indicated amounts of PX-478 were added after 24 hours, and proliferation rates and numbers of live cells were measured 72 hours post-treatment. (**A)** Proliferation rates of VERO or VERO-K cells measured using the MTS assay and expressed as fold changes compared to no PX-478 control cells. (**B**) Live cells were counted using trypan blue exclusion method and the numbers of live cells with 10μM or 20μM PX-478 were expressed as fold changes relative to 0μM PX-478. The numbers of live cells for VERO and VERO-K cells in the absence of PX-478 were 34x10^4^ and 54x10^4^ cells per mL, respectively. Error bars represent standard deviations from at least 3 independent experiments.
(TIF)

S6 Fig Levels of cMyc is unchanged by HIF-1α knockdown. **(A**) mRNA levels of cMyc in BCBL-1 cells after 48 hours in normoxia (N) or hypoxia (H), normalized to 18S internal control and expressed as fold changes compared to shScr cells in N. **(B**) Protein levels of cMyc in the nuclear lysates of BCBL-1 and BC-3 cells after 48 hours in N or H. β-actin is shown as a loading control.
(TIF)

S1 Table List of antibodies used in Western blot analysis. (TIF)

S2 Table List of primers used for RT-qPCR of mRNAs. (TIF)

S3 Table List of PrimePCR SYBR Green q-PCR Assays from Bio-Rad. (TIF)

S4 Table List of TaqMan MicroRNA Assays used for RT-qPCR of mature miRNAs. (TIF)
