# Supplementary figures and images for: Hypoxia-inducible factor-1 alpha as a therapeutic target for primary effusion lymphoma
Source: PLoS Pathog. 2017 Sep 18;13(9):e1006628. doi: 10.1371/journal.ppat.1006628 (PMC5619862; doi:10.1371/journal.ppat.1006628)

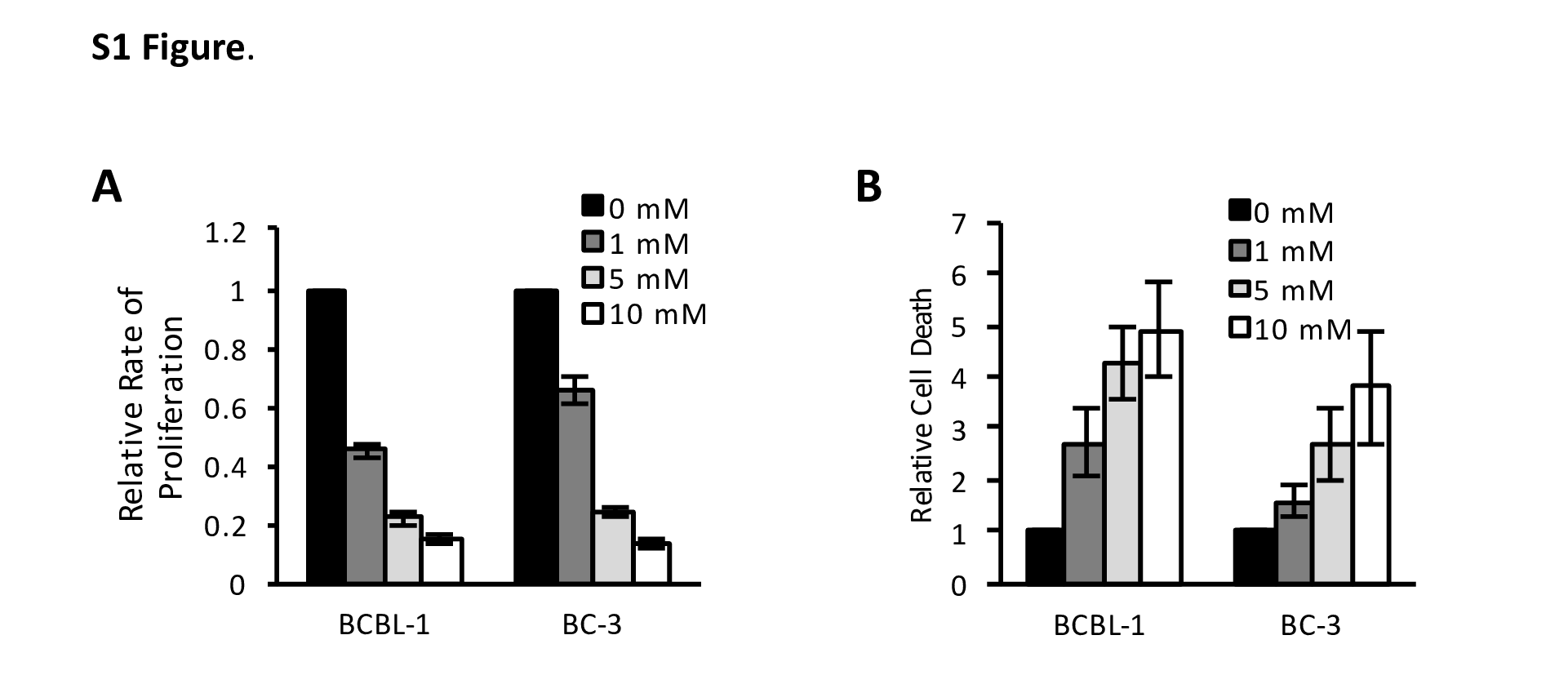

Supplement: S1 Fig — Cells were treated with various concentrations of glycolysis inhibitor 2-DG for 24 hours and rate of proliferation (A) and cell death (B) were measured using MTS assay and trypan blue exclusion method, respectively. Results are expressed as fold changes compared to no 2-DG control. Error bars represent standard deviations from at least 3 independent experiments. (TIF) [file ppat.1006628.s001.tif]

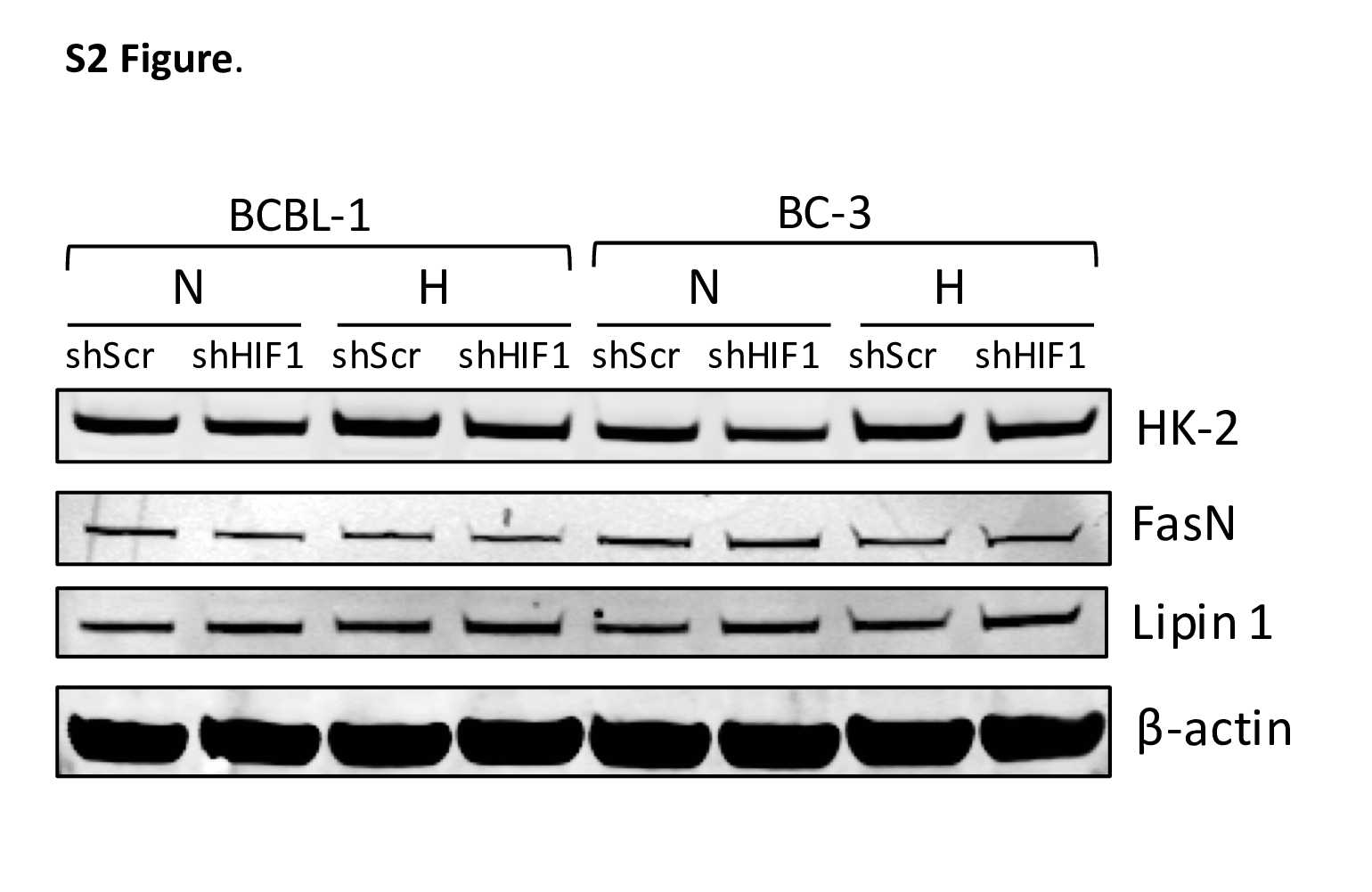

Supplement: S2 Fig — Protein levels of HK-2, Lipin 1, and FasN were measured in shScr and shHIF-1 cells after 48 hours in normoxia (N) or hypoxia (H). β-actin is shown as a loading control. (TIF) [file ppat.1006628.s002.tif]

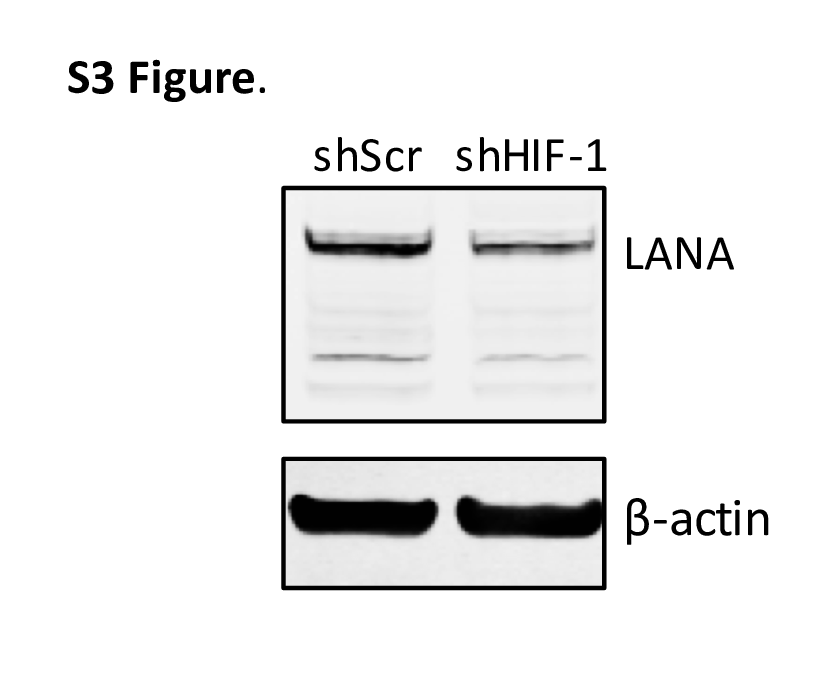

Supplement: S3 Fig — Western blot of LANA in the nuclear extracts of BCBL-1 cells cultured for 48 hours. β-actin is shown as a loading control. (TIF) [file ppat.1006628.s003.tif]

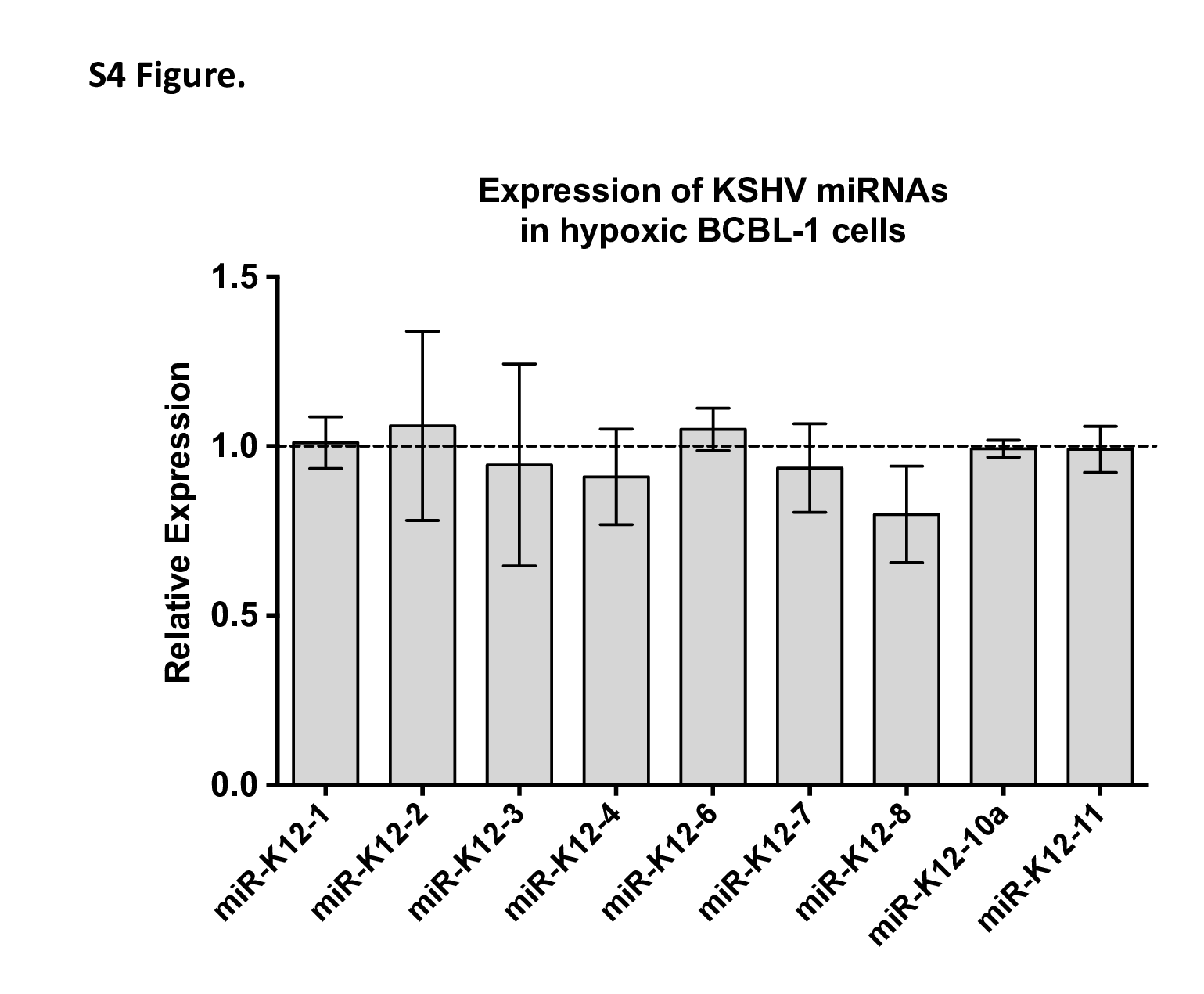

Supplement: S4 Fig — Levels of mature KSHV miRNAs were measured using taqman assays. The miRNA levels were normalized to that of RNU43 internal miRNA control and the results are expressed as fold change over normoxia. (TIF) [file ppat.1006628.s004.tif]

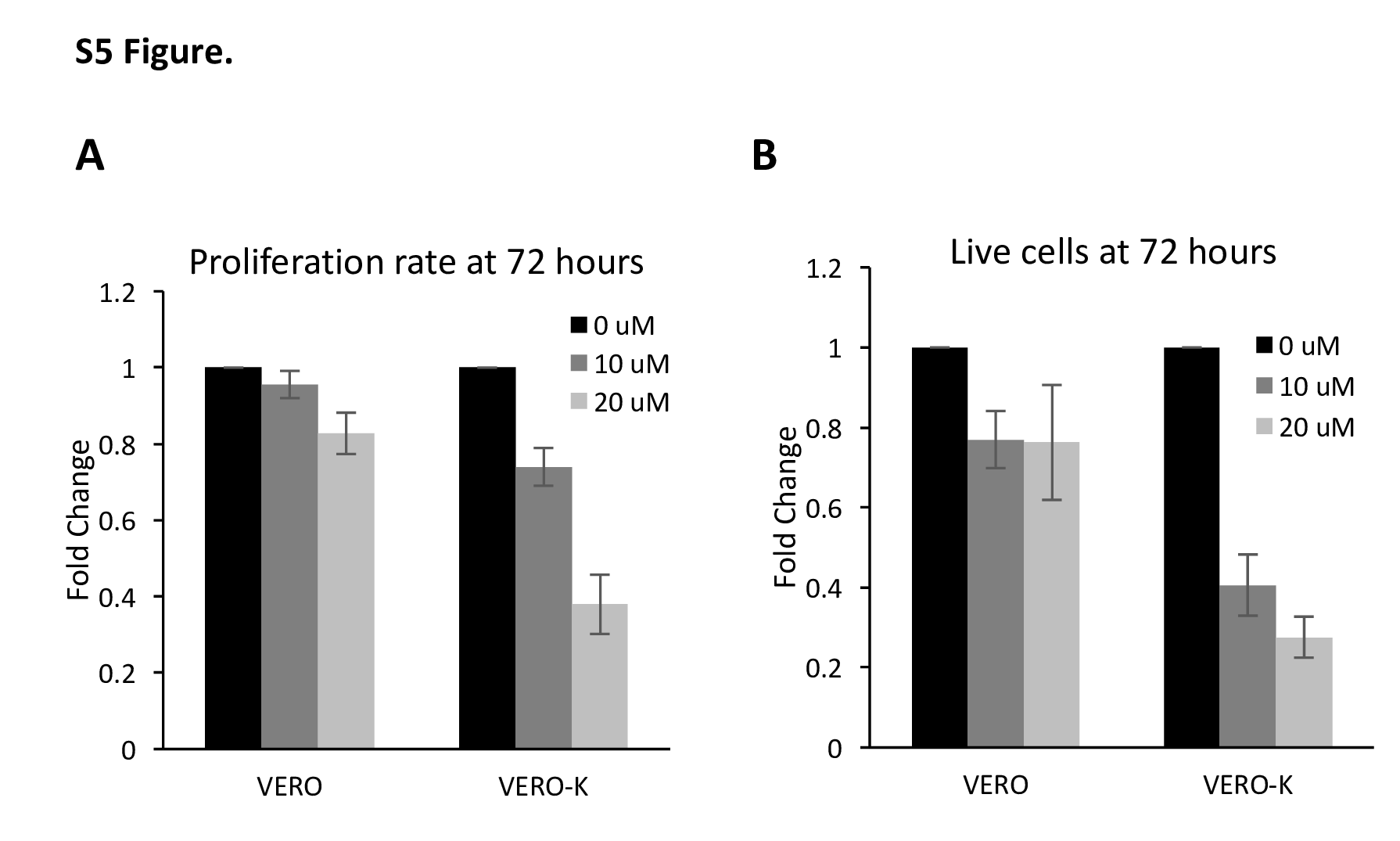

Supplement: S5 Fig — VERO and VERO-K cells were plated at 5x10^4^ cells per well of a 6-well plate. Indicated amounts of PX-478 were added after 24 hours, and proliferation rates and numbers of live cells were measured 72 hours post-treatment. (A) Proliferation rates of VERO or VERO-K cells measured using the MTS assay and expressed as fold changes compared to no PX-478 control cells. (B) Live cells were counted using trypan blue exclusion method and the numbers of live cells with 10μM or 20μM PX-478 were expressed as fold changes relative to 0μM PX-478. The numbers of live cells for VERO and VERO-K cells in the absence of PX-478 were 34x104 and 54x104 cells per mL, respectively. Error bars represent standard deviations from at least 3 independent experiments. (TIF) [file ppat.1006628.s005.tif]

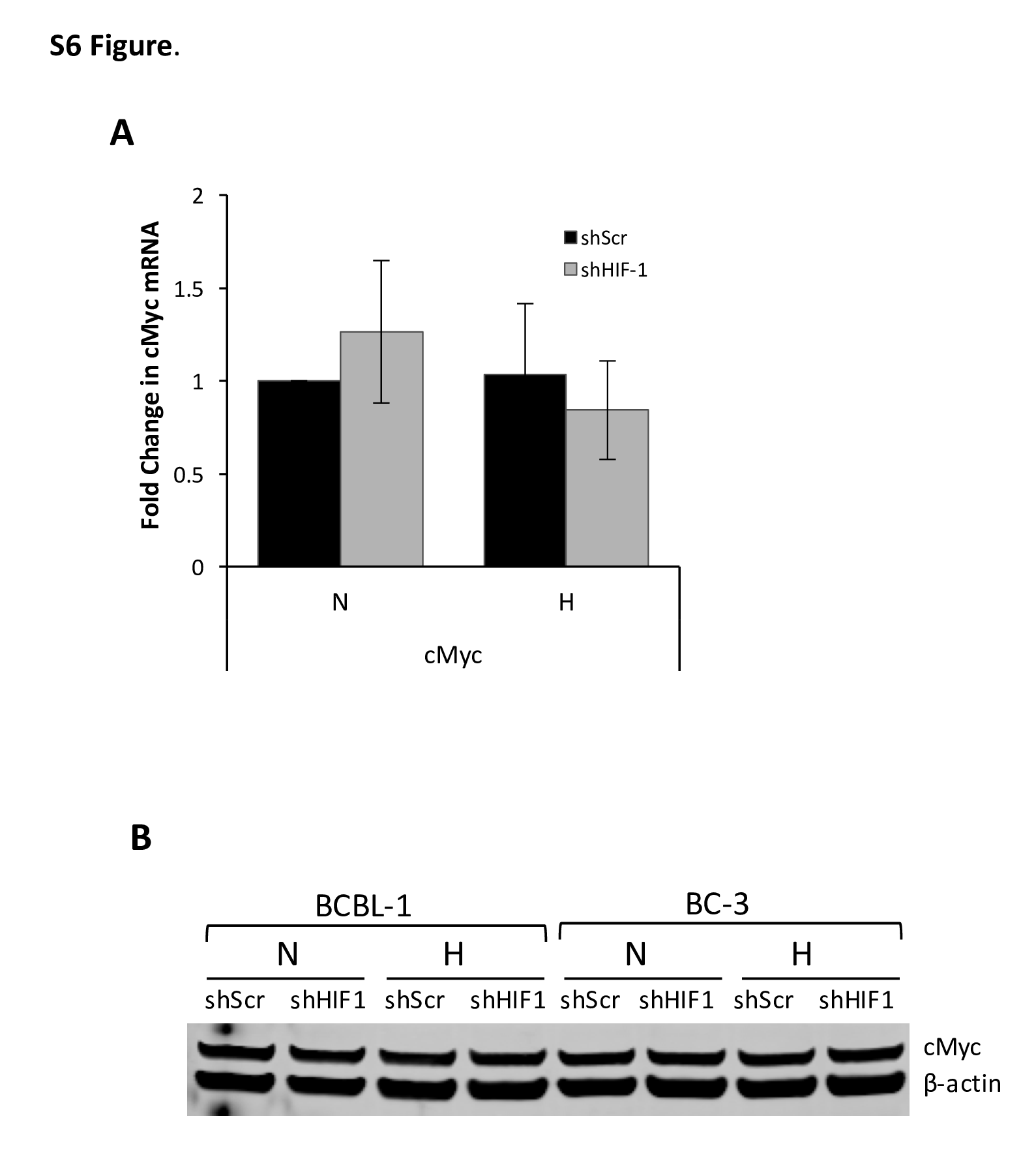

Supplement: S6 Fig — (A) mRNA levels of cMyc in BCBL-1 cells after 48 hours in normoxia (N) or hypoxia (H), normalized to 18S internal control and expressed as fold changes compared to shScr cells in N. (B) Protein levels of cMyc in the nuclear lysates of BCBL-1 and BC-3 cells after 48 hours in N or H. β-actin is shown as a loading control. (TIF) [file ppat.1006628.s006.tif]

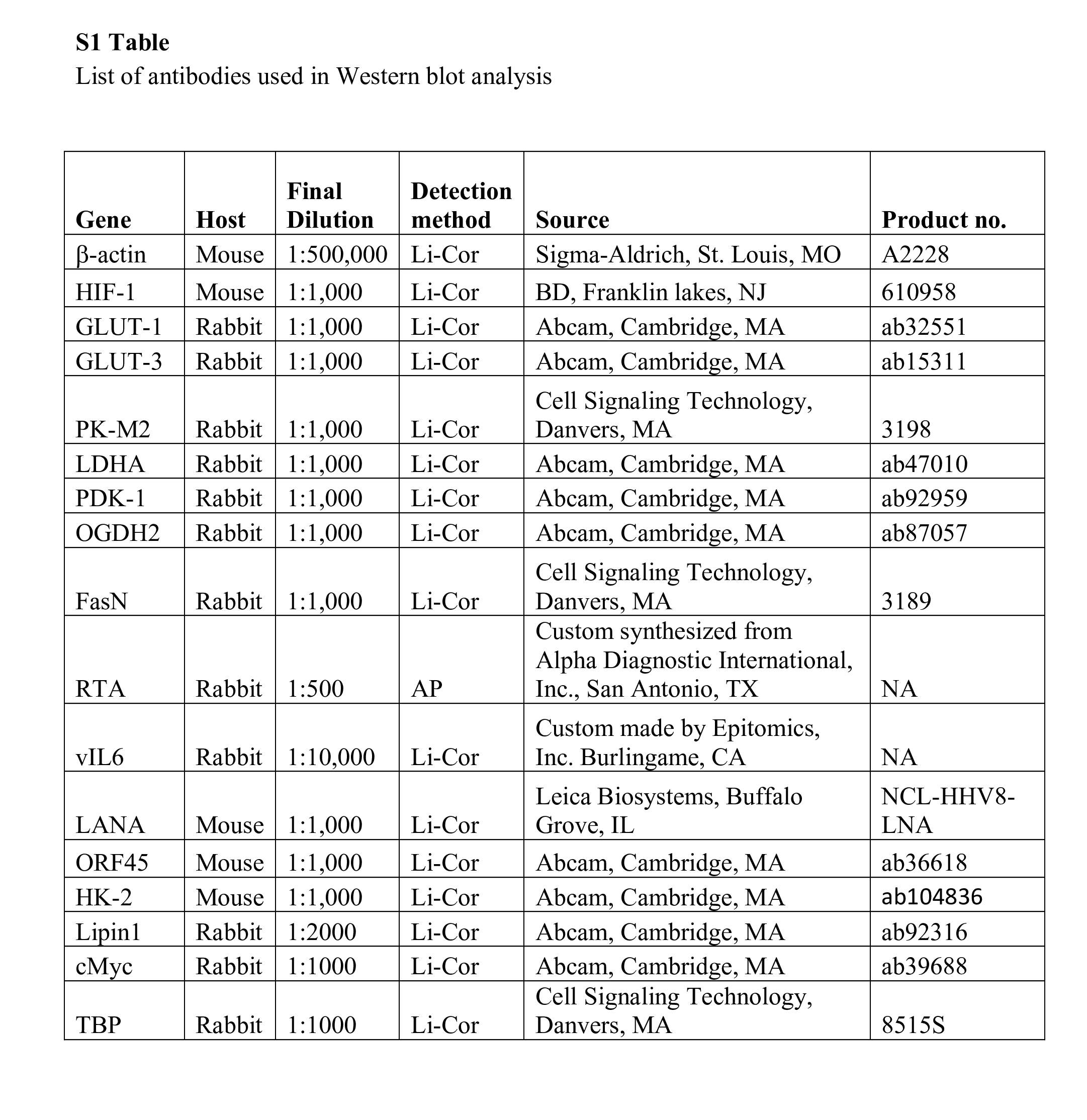

Supplement: S1 Table — (TIF) [file ppat.1006628.s007.tif]

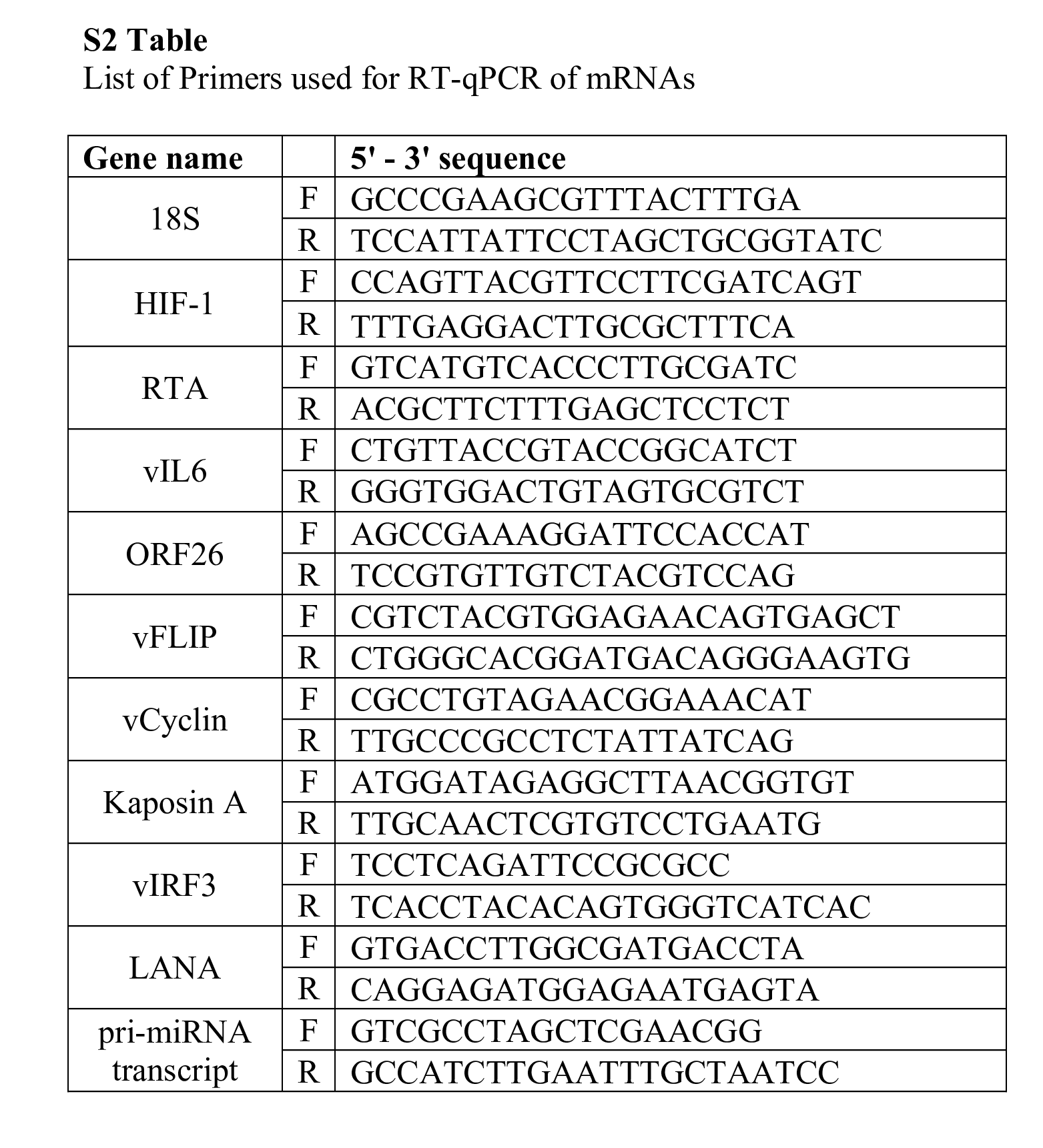

Supplement: S2 Table — (TIF) [file ppat.1006628.s008.tif]

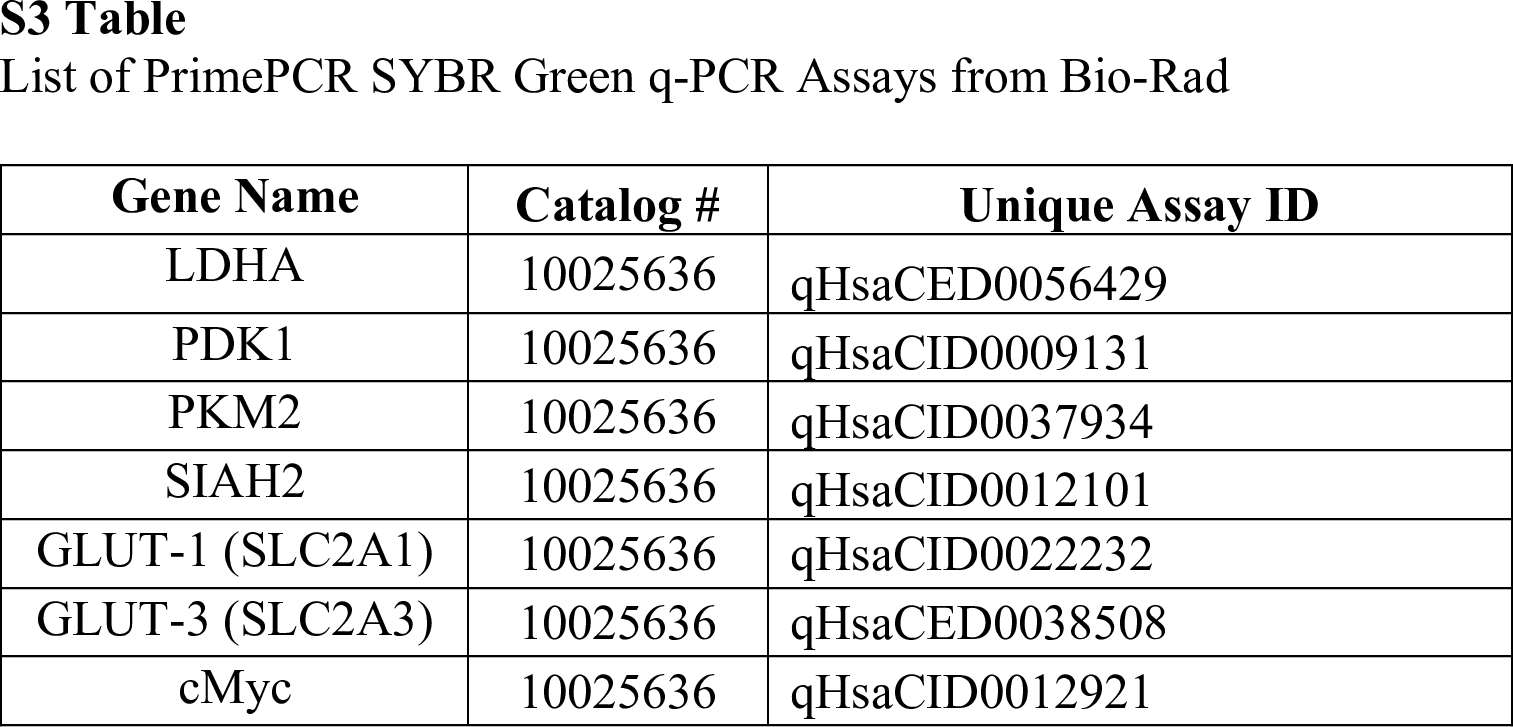

Supplement: S3 Table — (TIF) [file ppat.1006628.s009.tif]

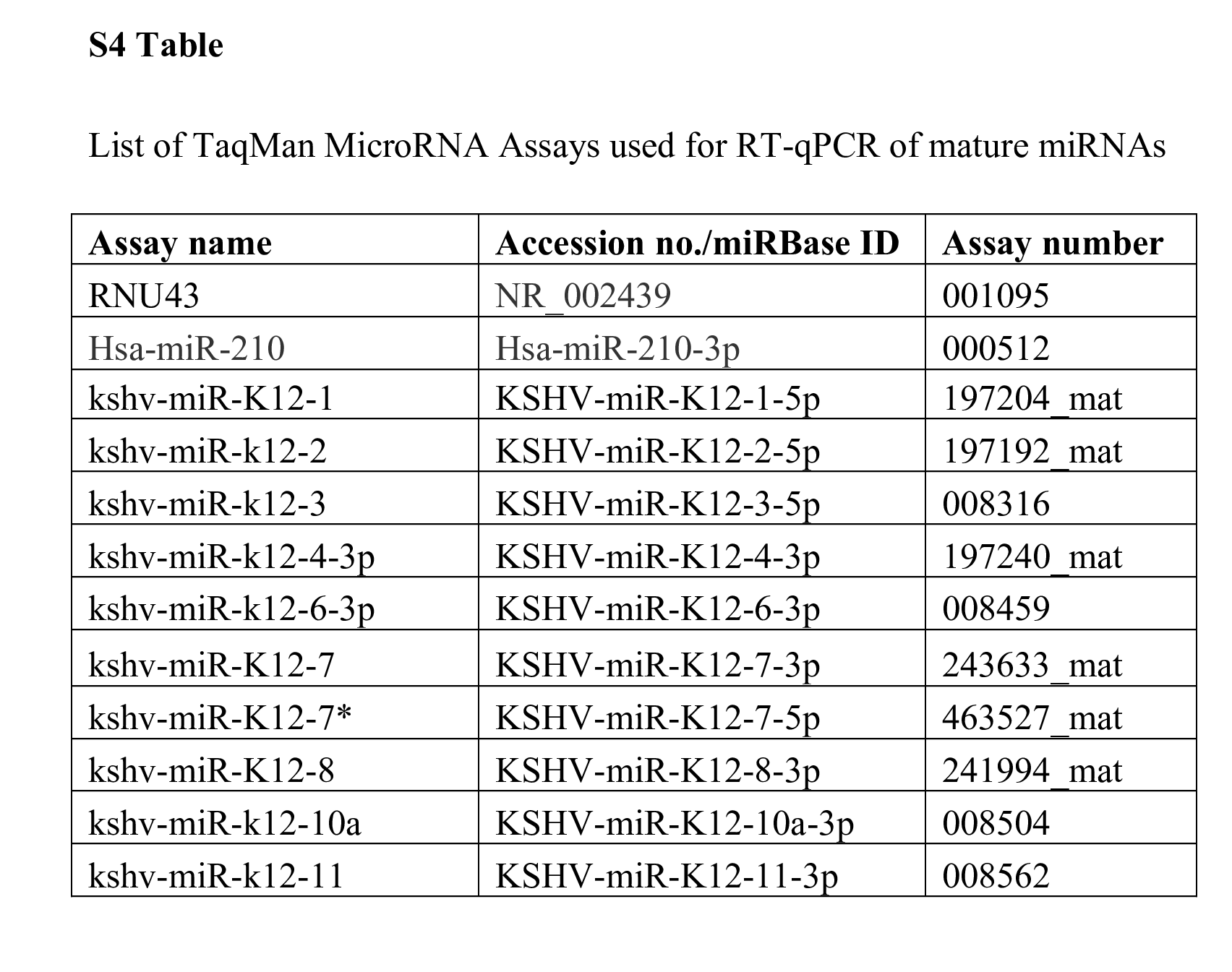

Supplement: S4 Table — (TIF) [file ppat.1006628.s010.tif]
